# A Survey of MicroRNA Length Variants Contributing to miRNome Complexity in Peach (*Prunus Persica L*.)

**DOI:** 10.3389/fpls.2012.00165

**Published:** 2012-07-26

**Authors:** Moreno Colaiacovo, Letizia Bernardo, Isabella Centomani, Cristina Crosatti, Lorenzo Giusti, Luigi Orrù, Gianni Tacconi, Antonella Lamontanara, Luigi Cattivelli, Primetta Faccioli

**Affiliations:** ^1^CRA Genomics Research Centre, Fiorenzuola d’ArdaItaly

**Keywords:** microRNA, isomiRs, next generation sequencing

## Abstract

MicroRNAs (miRNAs) are short non-coding RNA molecules produced from hairpin structures and involved in gene expression regulation with major roles in plant development and stress response. Although each annotated miRNA in miRBase (www.mirbase.org) is a single defined sequence with no further details on possible variable sequence length, isomiRs – namely the population of variants of miRNAs coming from the same precursors – have been identified in several species and could represent a way of broadening the regulatory network of the cell. Next-gen-based sequencing makes it possible to comprehensively and accurately assess the entire miRNA repertoire including isomiRs. The aim of this work was to survey the complexity of the peach miRNome by carrying out Illumina high-throughput sequencing of miRNAs in three replicates of five biological samples arising from a set of different peach organs and/or phenological stages. Three hundred-ninety-two isomiRs (miRNA and miRNA*-related) corresponding to 26 putative miRNA coding loci, have been highlighted by mirDeep-P and analyzed. The presence of the same isomiRs in different biological replicates of a sample and in different tissues demonstrates that the generation of most of the detected isomiRs is not random. The degree of mature sequence heterogeneity is very different for each individual locus. Results obtained in the present work can thus contribute to a deeper view of the miRNome complexity and to better explore the mechanism of action of these tiny regulators.

## Introduction

MicroRNAs (miRNAs) are short non-coding RNA molecules produced from hairpin structures and involved in gene expression regulation with major roles in plant development and stress response. MiRNAs are transcribed into a primary transcript which folds into a bulge with stem-loop conformation that is then cleaved by a Dicer-like (DCL) RNase III enzyme named DCL1. The cleavage results in a short duplex: one of the two strands forming the duplex and designated as miRNA* is then typically degraded while the other strand is incorporated into the RNA-induced silencing (RISC) complex where it mediates mRNA recognition and cleavage or translational repression (Jones-Rhoades et al., 2006; Voinnet, 2009; Xie et al., 2010).

Although each annotated miRNA in miRBase^1^ is a single defined sequence with no further details on possible variable sequence length, isomiRs – namely the population of variants of miRNAs coming from the same precursors – have been identified in several species and could represent a way of broadening the cell regulatory network (Ebhardt et al., 2009; Guo and Lu, 2010).

Vaucheret (2009) demonstrated the biological significance of mature miRNA length heterogeneity in *Arabidopsis* where the ath-miR168 can be processed as two different miRNA isoforms of 21 nt and 22 nt in length with different activities on AGO1 homeostasis (AGO1 is the Argonaute1 protein, a component of RISC complex that catalyzes broad miRNA- and siRNA-guided mRNA cleavage and translation repression Voinnet, 2009).

Alteration in miRNA end sequences can have strong effects on miRNA function due to the fact that the identity of the first 5′ nucleotide is the major determinant for AGO protein association (Takeda et al., 2008). As an example, Mi et al. (2008) found that AGO1 (which predominates in the miRNAs-mediated pathway) harbors miRNAs that favor a 5′ terminal uridine. A change at the 5′ terminal nucleotide of a miRNA predictably redirected it into a different AGO complex and altered its biological activity. Additionally, it was reported that the thermodynamic stability at the 5′ end of the strand is likely to affect the loading in the AGO complex (Eamens et al., 2009).

An accurate profile of the entire miRNA population of a biological sample provides useful information on miRNA activity and it can be used to compare the distribution of miRNA sequence variants in different samples. In fact, although the distribution of isomiRs across samples has been previously shown to be generally similar, examples in which the dominant isomiR is different from sample to sample have been found in animals (Lee et al., 2012) and could imply a functional role for specific isomiR sequences, besides affecting the accuracy and consistency of miRNA measurement.

This work aims to survey, by carrying out Illumina high-throughput sequencing, the complexity of peach miRNome through the analysis of the miRNA population of a set of samples representative of different tissues and developmental stages.

## Materials and Methods

### Plant material and RNA extraction

A 12-year-old tree grafted on wild seedling of the yellow-fleshed cv. Maycrest (*Prunus persica* (L.) Batsch), grown in Palazzolo di Sona, Verona, Italy (45.457°N, 10.822°E), was used as plant source material. Each sample was collected pooling together material from three different branches of the same plant. Four phenological stages (Chapman and Catlin, 1976) were considered: swollen bud, half-inch green, pink, bloom. Leaf and flower swollen buds were collected 41 days before flowering (DBF), half-inch leaves were collected 21 DBF, pink flower buds were collected six DBF. Codes were assigned to each samples: BF, pink; F, bloom; GF, swollen flower bud; O, half-inch green; GL, swollen leaf bud. Tissues were frozen in liquid nitrogen immediately after drawing. Total RNA was extracted from three independent samples with the Plant Total RNA Purification Kit (NORGEN Biotek Corp., Thorold, ON, Canada) following manufacturer instructions. RNA quality and concentration were evaluated with the Agilent 2100 Bioanalyzer RNA 6000 Nano assay (Agilent Technologies, Santa Clara, CA, USA).

### Small RNA libraries construction and sequencing

Preparation of small RNA libraries was performed with the TruSeq Small RNA Sample Prep Kit (Illumina, San Diego, CA, USA) following manufacturer instructions. Briefly, 1 μg of total RNA was ligated with adapters at 3′ and 5′ ends, without any size fractionation. Adapter-ligated RNA was reverse-transcribed with SuperScript II Reverse Transcriptase (Invitrogen, Carlsbad, CA, USA), then PCR-amplified (15 cycles). Samples were barcoded using 15 variants of the reverse primer provided with the kit. Libraries were pooled together and then purified on a 6% TBE PAGE gel after electrophoresis. Libraries quality and concentration were evaluated with the Agilent 2100 Bioanalyzer DNA 1000 assay. The obtained cDNAs were sequenced using the Illumina Genome Analyzer IIx.

### Data analysis

Reads were filtered with UEA sRNA plant toolkit^2^ (Moxon et al., 2008) to remove adaptor sequences, reads shorter than 18 nt or longer than 24, low-complexity reads, reads matching rRNAs or tRNAs and reads that did not match the peach genomic sequence available at “The International Peach Genome Initiative – www.rosaceae.org/peach/genome” (only those sequences with a full-length perfect match to the selected genome were retained). Reads from one replicate (randomly chosen) of each biological sample were then analyzed with the software miRDeep-P (Yang and Li, 2011, default parameters) to identify miRNA loci expressed in all the five tested samples (GF, GL, B, F, O). Reads associated to these loci were then also screened in the remaining two replicates.

Read counts for each variant were divided by the total number of reads with a match in the peach genome in each sample and normalized to 1,000,000 reads. Reads that could be related to more than one locus were assigned by MiRDeep-P to all possible involved loci.

IsomiRs for each putative locus were blasted against miRBase (release 18) to search for the loci related to previously known miRNAs (Kozomara and Griffith-Jones, 2011). Blast vs. mature sequences was based on the following parameters: outfmt 6, task blastn, dust “no,” *e*-value 10, word_size 7, reward 2, num_alignments 10. Blast vs. precursor sequences was based on the following parameters: outfmt 6, task blastn, dust “no,” *e*-value 10e−3, num_alignments 10.

The correlation between biological replicates was evaluated by calculating the Pearson coefficient for all the possible pairs of replicates belonging to the same biological sample, as well as samples from different tissues for sake of comparison. We decided to remove from the set a sequence (HE860285) whose expression level was abnormally high, because its presence caused the Pearson correlation to be almost one in every comparison, irrespective of the tissue.

To identify miRNAs isomiRs that were differentially expressed among the biological samples, a *t*-test was performed for all the possible comparisons. An isomiR was considered as differentially expressed in a specific comparison if its *p*-value was less than 0.05. The whole set of reads associated with the miRNA loci was then used to perform a hierarchical clustering with R software, by applying the Canberra metric to calculate the distances between the expression vectors of the samples across the reads.

MiRNA target identification was carried out by psRNATarget^3^ (Dai and Zhao, 2011), with default parameters. To score the complementarity between small RNA and their target transcript, psRNATarget applies the scoring schema of miRU by Zhang (2005). The *maximum expectation* is the threshold of the score. A small RNA/target site pair will be discarded if its score is greater than the threshold. The default cut-off threshold is 3.0.

The accessibility of the mRNA target site to small RNA has been identified as one of the important factors involved in target recognition because the secondary structure (stem, etc.) around the target site will prevent the small RNA and mRNA target from contacting. The psRNATarget server employes RNAup to calculate target accessibility, which is represented by the energy required to open (unpair) secondary structure around the target site (usually the complementary region with small RNA and up/downstream) on target mRNA. The lower the energy the higher the possibility that small RNA is able to contact (and cleave) target mRNA. PsRNATarget uses a software, namely RNAup, described by Mückstein et al. (2006) to calculate this value, denoted as UPE.

All the miRNA-related sequences were submitted to the EMBL database, whereas the sequencing raw reads were submitted to the NCBI SRA (BioProject accession: PRJNA167962).

## Results

### Sequencing peach small RNA libraries

Illumina deep sequencing was used to profile the whole miRNA set of five different samples corresponding to different organs and/or phenological stages. Three replicates were analyzed for each sample. A total number of 40,764,330 sequence reads were obtained and filtered as reported in Section “Materials and Methods.” Details on the results of each filtering step are reported in Table 1. On average, 2,717,622 raw reads and 664,777 clean reads perfectly matching the genome were obtained in each of the 15 samples.

**Table 1:** **Reports the number of filtered reads perfectly matching the peach genome in each of the tested samples**.

Sample	Raw reads	Matching adaptors	Matching adaptors (18–24 nt)	Low-complexity filtered (non-redundant)	rRNA/tRNA removed (non-redundant)	Matching peach genome (non-redundant)
BF1	2842653	2332661	1592671	1582689 (356161)	1335567 (333478)	797297 (207233)
BF2	2553116	2124140	1570583	1560824 (370103)	1401892 (351620)	817438 (220223)
BF3	2641037	2184480	1646360	1635958 (387915)	1466005 (368373)	853043 (235873)
F1	2523898	1819981	1180424	1173273 (215571)	858130 (194546)	368958 (78332)
F2	2898014	2361457	1389585	1381108 (233414)	1040918 (214222)	630870 (115644)
F3	3613383	2923242	1768783	1757830 (320484)	1362239 (297258)	700729 (143030)
GF1	2696289	2170904	1354915	1346476 (307349)	1200810 (289976)	774146 (190043)
GF2	3722325	2848155	1962881	1950819 (429497)	1570664 (405303)	928251 (248399)
GF3	2952035	2254276	1327544	1319295 (308967)	1145330 (291401)	609728 (168939)
GL1	2357377	1707677	1072569	1065997 (241873)	870779 (220909)	481523 (122824)
GL2	2304406	1729282	1080322	1073580 (253835)	929602 (234995)	460632 (125267)
GL3	1822754	1345521	745352	740785 (207198)	616318 (190449)	338168 (111111)
O1	3704334	2907197	1811057	1799817 (327514)	1384994 (302970)	890186 (182703)
O2	1896816	1509747	1068609	1061991 (226499)	911190 (208620)	624084 (129672)
O3	2235893	1753142	1286833	1278775 (282053)	1093002 (263673)	696601 (162637)

*BF, pink; F, bloom; GF, swollen flower bud; O, half-inch green; GL, swollen leaf bud*.

One technical replicate (randomly chosen and numbered as “1”) of each sample was subsequently analyzed with miRDeeP-P which highlighted the putative miRNA coding loci of the peach genome expressed in the five tested samples (reported in Files S1–S5 in Supplementary Material and summarized in File S6 in Supplementary Material). Twenty-six putative miRNA coding loci were expressed in all samples according to miRDeep-P results. The length of the putative precursors was between 41 nt and 227 nt (average length of 104 nt), while average mature miRNAs size was 22 nt.

These 26 miRNAs were selected and, for each of them, the corresponding associated reads were searched in all the replicates of each sample. The results (miRNAs and miRNAs* associated reads) are reported in Table 2 for each locus, the link between locus name and locus position can be found in File S7 in Supplementary Material and retrieved at www.rosaceae.org/peach/genome.

**Table 2:** **Reports the read count (divided by the total number of reads with a perfect match to the peach genome and normalized to 1,000,000 reads) of 26 putative miRNA coding loci that were expressed in all the 15 samples according to miRDeep-P results**.

miRNA	Reads	EMBL accession number	BF1	BF2	BF3	F1	F2	F3	GF1	GF2	GF3	GL1	GL2	GL3	O1	O2	O3
1_10	AGTTTGTGCGTGAATCGAACC	HE862997	2.5	1.2	1.2	35.2	11.1	45.7	2.6	4.3	4.9	0	10.9	5.9	1.1	0	0
	CAGTTTGTGCGTGAATCGAAC	HE860429	6.3	9.8	19.9	24.4	7.9	20	3.9	10.8	14.8	8.3	17.4	5.9	3.4	0	8.6
	TTAGATTCACGCACAAAC	HE862999	0	0	0	0	0	0	1.3	2.2	1.6	0	0	0	0	0	0
	TTAGATTCACGCACAAACT	HE860429	3.8	6.1	4.7	5.4	0	4.3	3.9	4.3	3.3	2.1	6.5	0	1.1	1.6	0
	TTAGATTCACGCACAAACTC	HE863001	2.5	6.1	4.7	8.1	0	5.7	1.3	4.3	1.6	0	4.3	0	4.5	3.2	4.3
	TTAGATTCACGCACAAACTCG	HE860293	66.5	93	83.2	208.7	71.3	125.6	67.2	106.7	132.8	105.9	125.9	230.7	27	40.1	58.9
1_15	AACCACAAATCTCTTGGACTCCTG	HE860430	1.3	0	1.2	0	0	0	1.3	1.1	0	0	0	0	0	1.6	0
	AAGAGATTTGTGGTTACTCAC	HE863003	0	0	1.2	0	0	0	1.3	2.2	1.6	0	4.3	3	0	1.6	1.4
	AAGAGATTTGTGGTTACTCACC	HE860430	2.5	2.4	0	0	0	1.4	0	1.1	0	0	0	0	0	0	0
	AAGAGATTTGTGGTTACTCACCG	HE863005	0	7.3	1.2	0	0	0	2.6	1.1	1.6	0	0	0	1.1	0	2.9
	AAGAGATTTGTGGTTACTCACCGT	HE860431	12.5	18.4	12.9	5.4	3.2	2.9	14.2	9.7	6.6	4.2	2.2	0	6.7	6.4	7.2
	AGAGATTTGTGGTTACTCAC	HE863007	6.3	2.4	3.5	2.7	0	0	0	1.1	0	2.1	0	0	1.1	1.6	1.4
	AGAGATTTGTGGTTACTCACCG	HE860431	1.3	4.9	4.7	0	1.6	0	2.6	0	0	2.1	0	3	1.1	4.8	4.3
	AGAGATTTGTGGTTACTCACCGT	HE863009	0	1.2	2.3	0	1.6	0	0	1.1	1.6	0	0	0	2.2	0	2.9
	AGAGATTTGTGGTTACTCACCGTT	HE860297	117.9	162.7	126.6	27.1	36.5	25.7	71	65.7	49.2	45.7	43.4	20.7	80.9	52.9	34.5
	ATTTACATCCAACGGTGAGTAACC	HE860432	0	0	0	0	0	0	0	0	0	2.1	2.2	0	0	0	0
	CAAGAGATTTGTGGTTACTCA	HE863011	0	0	0	0	1.6	0	0	0	0	0	0	3	1.1	0	0
	CAAGAGATTTGTGGTTACTCACC	HE860432	1.3	0	1.2	0	0	0	0	2.2	0	0	0	0	1.1	0	0
	CAAGAGATTTGTGGTTACTCACCG	HE863013	0	2.4	2.3	2.7	3.2	4.3	2.6	2.2	0	0	0	0	0	0	0
	CCAAGAGATTTGTGGTTACTCA	HE860433	0	0	0	0	0	0	0	0	0	0	0	0	1.1	0	0
	TCCAAGAGATTTGTGGTTACTCAC	HE863015	1.3	0	0	0	0	1.4	0	0	0	2.1	0	0	0	0	1.4
1_25	CGAAACCTCCCATTCCAA	HE860433	1.3	0	0	0	0	0	1.3	2.2	0	2.1	2.2	3	0	0	0
	GAGAGGTTGCCGGAAAGA	HE863017	0	0	0	0	0	0	0	0	0	2.1	0	0	0	0	0
	GGGTGAGAGGTTGCCGGAAA	HE860434	0	0	2.3	0	0	0	0	0	0	2.1	0	0	0	1.6	4.3
	GGGTGAGAGGTTGCCGGAAAG	HE863019	2.5	9.8	16.4	0	0	0	10.3	14	8.2	12.5	15.2	11.8	15.7	30.4	38.8
	GGGTGAGAGGTTGCCGGAAAGA	HE860434	32.6	24.5	52.8	0	3.2	0	96.9	206.8	157.4	105.9	121.6	106.5	75.3	91.3	208.2
	GGGTGAGAGGTTGCCGGAAAGAA	HE863021	0	0	0	0	0	0	1.3	0	0	0	0	3	0	0	0
	GGTGAGAGGTTGCCGGAAAGAAT	HE860435	0	0	0	0	0	0	0	0	0	4.2	0	0	0	0	0
	TCCGAAACCTCCCATTCCAA	HE863023	1.3	0	1.2	0	0	1.4	3.9	0	0	0	0	0	2.2	0	1.4
	TCCGAAACCTCCCATTCCAAT	HE860435	3.8	1.2	1.2	0	0	0	1.3	1.1	0	0	0	0	0	0	1.4
	TCCGAAACCTCCCATTCCAATG	HE863025	0	0	0	0	0	0	3.9	0	0	0	0	0	0	0	0
	TTCCGAAACCTCCCATTCCAA	HE860436	17.6	30.6	17.6	5.4	6.3	15.7	29.7	49.6	27.9	33.2	39.1	26.6	13.5	11.2	8.6
	TTCCGAAACCTCCCATTCCAAT	HE863027	1.3	2.4	1.2	2.7	0	1.4	6.5	1.1	3.3	0	2.2	3	0	3.2	0
	TTGGGTGAGAGGTTGCCGGAA	HE860436	0	0	0	0	0	0	0	0	0	0	0	0	1.1	0	0
	TTGGGTGAGAGGTTGCCGGAAA	HE863029	2.5	0	0	0	0	2.9	1.3	0	0	0	0	0	0	0	0
	TTTCCGAAACCTCCCATT	HE860437	2.5	2.4	1.2	0	0	1.4	0	2.2	1.6	0	4.3	3	1.1	0	0
	TTTCCGAAACCTCCCATTC	HE863031	3.8	1.2	4.7	0	0	8.6	7.8	5.4	3.3	2.1	4.3	3	1.1	0	0
	TTTCCGAAACCTCCCATTCC	HE860437	8.8	8.6	23.4	5.4	9.5	17.1	34.9	23.7	23	10.4	21.7	3	2.2	4.8	4.3
	TTTCCGAAACCTCCCATTCCA	HE863033	15.1	11	12.9	0	4.8	5.7	29.7	22.6	13.1	16.6	21.7	3	1.1	0	5.7
	TTTCCGAAACCTCCCATTCCAA	HE860304	1135.1	1196.4	1134.8	401.1	391.5	687.9	1802	2047.9	1835.2	1277.2	1417.6	777.7	433.6	387.8	446.5
	TTTCCGAAACCTCCCATTCCAAT	HE860438	18.8	15.9	16.4	8.1	6.3	12.8	14.2	21.5	14.8	20.8	28.2	3	3.4	4.8	5.7
1_26	AAAAAGACTCAACAACCCATGTTT	HE863035	0	0	0	0	0	0	0	0	0	2.1	0	0	0	0	0
	AAAAGACTCAACAACCCATGT	HE860438	0	1.2	1.2	2.7	0	1.4	0	0	0	0	2.2	0	0	1.6	0
	AAAAGACTCAACAACCCATGTTT	HE863037	0	0	0	0	0	0	0	0	0	2.1	0	0	0	0	0
	AAAGACTCAACAACCCATGT	HE860439	1.3	0	0	0	0	0	0	0	0	0	0	0	0	0	0
	AAAGGCATAGTAGGGTTTAGGA	HE863039	0	0	0	0	0	0	1.3	0	0	0	0	0	0	0	1.4
	AAAGGCATAGTAGGGTTTAGGAAG	HE860439	3.8	0	1.2	0	0	0	6.5	8.6	3.3	2.1	4.3	0	0	0	1.4
	AAGGCATAGTAGGGTTTAGGAAGT	HE863041	1.3	0	0	0	0	0	0	0	0	0	0	0	0	0	0
	ACCCCGCCCATTCCAAATATT	HE860440	0	0	0	2.7	0	1.4	1.3	0	0	0	0	0	0	1.6	0
	ACCCCGCCCATTCCAAATATTT	HE863043	0	0	0	0	0	1.4	1.3	0	0	0	0	0	0	0	0
	ATATTTTCTAAGCCTACTGTC	HE860440	7.5	3.7	5.9	8.1	3.2	8.6	22	20.5	26.2	16.6	13	11.8	13.5	4.8	7.2
	CAAATATTTTCTAAGCCTACTGTC	HE863045	0	0	0	2.7	0	0	0	0	0	0	0	0	0	0	0
	CATAGTAGGGTTTAGGAA	HE860441	0	0	0	0	0	0	1.3	0	0	2.1	0	0	0	0	0
	CATAGTAGGGTTTAGGAAGTT	HE863047	0	0	0	0	0	0	1.3	0	0	0	0	0	0	0	0
	CATAGTAGGGTTTAGGAAGTTT	HE860441	0	0	2.3	0	0	1.4	0	0	0	2.1	0	0	0	0	0
	CATAGTAGGGTTTAGGAAGTTTT	HE863049	1.3	1.2	2.3	0	1.6	2.9	5.2	3.2	1.6	2.1	0	0	1.1	0	2.9
	CATAGTAGGGTTTAGGAAGTTTTT	HE860442	7.5	7.3	4.7	5.4	3.2	4.3	10.3	11.9	3.3	10.4	0	3	2.2	1.6	4.3
	CTTTGCCAACCCCGCCCATTCC	HE863051	2.5	0	0	0	1.6	2.9	6.5	5.4	3.3	0	0	0	2.2	0	1.4
	CTTTGCCAACCCCGCCCATTCCA	HE860442	1.3	0	0	0	0	0	0	0	1.6	0	0	0	0	0	0
	CTTTGCCAACCCCGCCCATTCCAA	HE863053	0	1.2	1.2	0	0	0	5.2	1.1	4.9	16.6	2.2	8.9	1.1	0	1.4
	GAAAGGCATAGTAGGGTTTAGGA	HE860443	1.3	0	1.2	0	0	0	0	1.1	0	2.1	0	0	1.1	1.6	0
	GAAAGGCATAGTAGGGTTTAGGAA	HE863055	1.3	0	5.9	2.7	0	5.7	3.9	6.5	0	0	0	0	0	1.6	4.3
	GCCAACCCCGCCCATTCCAA	HE860443	0	0	0	0	0	0	0	0	0	0	0	0	1.1	0	0
	GGAATGAGCGTGTTGGAAA	HE863057	1.3	0	1.2	0	0	0	0	0	0	0	0	0	0	0	1.4
	GGAATGAGCGTGTTGGAAAA	HE860444	1.3	1.2	0	0	0	0	1.3	0	0	0	2.2	0	1.1	1.6	1.4
	GGAATGAGCGTGTTGGAAAAG	HE863059	7.5	4.9	3.5	2.7	3.2	4.3	5.2	8.6	4.9	10.4	6.5	3	5.6	3.2	12.9
	GGAATGAGCGTGTTGGAAAAGA	HE860444	2.5	4.9	5.9	0	0	0	9	2.2	8.2	29.1	15.2	11.8	0	1.6	0
	GGAATGAGCGTGTTGGAAAAGAA	HE863061	0	0	0	0	0	0	1.3	0	0	2.1	0	0	1.1	0	0
	TATTTTCTAAGCCTACTGTC	HE860445	0	0	1.2	0	0	0	0	3.2	0	4.2	0	0	0	0	0
	TCTAAGCCTACTGTCTTTCCC	HE863063	0	0	0	0	0	2.9	2.6	1.1	0	2.1	0	0	2.2	1.6	0
	TCTAAGCCTACTGTCTTTCCCT	HE860445	0	0	0	0	0	1.4	1.3	0	0	0	0	0	1.1	1.6	0
	TGCCAACCCCGCCCATTCCA	HE863065	1.3	0	0	0	0	1.4	0	0	0	0	0	0	0	0	0
	TGCCAACCCCGCCCATTCCAA	HE860446	6.3	1.2	2.3	0	0	4.3	2.6	2.2	1.6	6.2	2.2	5.9	2.2	3.2	1.4
	TGCCAACCCCGCCCATTCCAAA	HE863067	6.3	4.9	2.3	5.4	0	4.3	10.3	11.9	16.4	14.5	28.2	8.9	6.7	8	7.2
	TGGAATGAGCGTGTTGGAAAA	HE860446	1.3	0	0	0	0	0	0	1.1	0	0	0	0	0	0	0
	TTCTTTGCCAACCCCGCCCATT	HE863069	1.3	0	0	0	0	1.4	2.6	0	1.6	4.2	4.3	3	1.1	0	0
	TTGCCAACCCCGCCCATT	HE860447	1.3	1.2	0	0	0	1.4	0	2.2	4.9	2.1	0	0	0	0	0
	TTGCCAACCCCGCCCATTC	HE863071	3.8	2.4	2.3	2.7	0	0	7.8	10.8	6.6	4.2	2.2	0	1.1	0	0
	TTGCCAACCCCGCCCATTCC	HE860447	26.3	24.5	15.2	10.8	4.8	17.1	71	40.9	78.7	35.3	26.1	53.2	10.1	24	12.9
	TTGCCAACCCCGCCCATTCCA	HE863073	5	3.7	3.5	5.4	0	1.4	10.3	5.4	9.8	2.1	8.7	5.9	3.4	6.4	5.7
	TTGCCAACCCCGCCCATTCCAA	HE860305	180.6	163.9	138.3	140.9	85.6	119.9	260.9	266.1	477.3	344.7	382.1	275	126.9	203.5	189.5
	TTGCCAACCCCGCCCATTCCAAA	HE860448	0	0	2.3	0	1.6	1.4	2.6	3.2	3.3	0	2.2	0	1.1	1.6	2.9
	TTGCCAACCCCGCCCATTCCAAAT	HE863075	1.3	0	0	0	0	0	0	0	0	0	0	0	0	1.6	0
	TTTGAAGCAGATGATGGAAC	HE860448	0	0	0	0	0	0	1.3	0	0	0	0	0	0	0	0
	TTTGCCAACCCCGCCCAT	HE863077	0	2.4	0	0	0	0	1.3	0	3.3	0	2.2	0	1.1	0	1.4
	TTTGCCAACCCCGCCCATT	HE860449	2.5	0	0	2.7	0	0	6.5	0	8.2	2.1	2.2	0	0	6.4	0
	TTTGCCAACCCCGCCCATTC	HE863079	5	1.2	1.2	2.7	0	1.4	3.9	2.2	8.2	4.2	0	3	2.2	4.8	1.4
	TTTGCCAACCCCGCCCATTCC	HE860449	20.1	19.6	14.1	10.8	7.9	12.8	67.2	26.9	73.8	33.2	41.2	50.3	15.7	19.2	7.2
	TTTGCCAACCCCGCCCATTCCA	HE863081	5	8.6	3.5	2.7	1.6	1.4	22	9.7	13.1	10.4	13	5.9	5.6	3.2	5.7
	TTTGCCAACCCCGCCCATTCCAA	HE860450	115.4	71	70.3	59.6	36.5	75.6	148.6	136.8	239.5	240.9	223.6	171.5	57.3	134.6	83.3
	TTTGCCAACCCCGCCCATTCCAAA	HE863083	0	1.2	1.2	0	0	0	3.9	2.2	11.5	0	4.3	11.8	0	0	0
1_29	AGGTGGGCATACTGCCAACTG	HE860450	3.8	2.4	2.3	13.6	4.8	10	1.3	0	0	0	0	0	3.4	1.6	1.4
	ATTGGCATTCTGTCCACCTCC	HE863085	0	1.2	0	0	1.6	0	0	0	0	0	0	0	1.1	0	0
	TGGCATTCTGTCCACCTCC	HE860451	1.3	0	0	0	0	1.4	0	0	0	0	2.2	0	0	0	0
	TTGGCATTCTGTCCACCT	HE863087	6.3	6.1	7	13.6	11.1	7.1	2.6	1.1	1.6	2.1	0	5.9	0	0	0
	TTGGCATTCTGTCCACCTC	HE860451	18.8	24.5	15.2	13.6	28.5	27.1	1.3	5.4	8.2	2.1	4.3	0	0	1.6	8.6
	TTGGCATTCTGTCCACCTCC	HE860307	89.1	121.1	89.1	127.4	141.1	225.5	80.1	53.9	59	27	26.1	20.7	20.2	35.3	25.8
	TTGGCATTCTGTCCACCTCCT	HE863089	36.4	29.4	36.3	10.8	26.9	62.8	23.3	15.1	13.1	2.1	4.3	3	4.5	4.8	1.4
	TTGGCATTCTGTCCACCTCCTC	HE860452	1.3	0	0	0	0	0	0	0	0	0	0	0	0	0	0
1_3	AACATGATCATCCGAATGAT	HE863091	0	0	0	0	0	0	0	0	0	0	0	0	1.1	0	0
	AATGCTGTCTGGTTCGAGA	HE860452	1.3	2.4	1.2	0	1.6	2.9	0	1.1	3.3	2.1	2.2	0	0	1.6	1.4
	ACCAGGCTTCATTCCCCC	HE863093	1.3	0	0	0	0	0	0	0	0	0	0	0	0	0	0
	ATCCGAATGATCTCGGACCAGGCT	HE860453	0	0	0	0	0	0	0	0	0	0	0	0	1.1	0	0
	ATCTCGGACCAGGCTTCATTCCCC	HE863095	6.3	14.7	17.6	0	9.5	11.4	2.6	1.1	0	6.2	6.5	0	1.1	4.8	5.7
	ATGCTGTCTGGTTCGAGA	HE860453	0	0	0	2.7	0	0	0	0	0	0	0	0	0	0	0
	CGGACCAGGCTTCATTCC	HE863097	0	0	0	0	0	0	0	1.1	0	0	2.2	0	2.2	0	0
	CGGACCAGGCTTCATTCCC	HE860454	1.3	2.4	0	2.7	1.6	0	1.3	5.4	1.6	0	0	3	1.1	0	0
	CGGACCAGGCTTCATTCCCC	HE863099	282.2	208	289.6	336.1	187	182.7	235.1	213.3	203.4	265.8	251.8	174.5	449.3	299.6	328.7
	CGGACCAGGCTTCATTCCCCC	HE860454	1.3	1.2	0	2.7	3.2	1.4	1.3	1.1	0	2.1	4.3	0	0	1.6	0
	CTCGGACCAGGCTTCATTCC	HE863101	0	2.4	1.2	2.7	1.6	1.4	1.3	0	1.6	0	0	0	1.1	0	0
	CTCGGACCAGGCTTCATTCCC	HE860455	66.5	83.2	65.6	132.8	42.8	129.9	9	9.7	9.8	10.4	17.4	17.7	16.9	46.5	43.1
	CTCGGACCAGGCTTCATTCCCC	HE863103	151.8	165.2	175.8	192.4	130	189.8	165.3	159.4	146	126.7	147.6	136	75.3	187.5	183.7
	CTCGGACCAGGCTTCATTCCCCC	HE860455	0	0	0	0	0	1.4	1.3	0	0	2.1	0	0	0	0	0
	GAATGCTGTCTGGTTCGAGAC	HE863105	3.8	1.2	0	5.4	0	1.4	0	0	0	0	0	0	1.1	1.6	0
	GACCAGGCTTCATTCCCC	HE860456	1.3	0	0	0	0	0	0	0	1.6	0	0	0	0	0	0
	GGAATGCTGTCTGGTTCGA	HE863107	0	0	0	0	0	0	1.3	0	0	0	0	0	0	0	0
	GGAATGCTGTCTGGTTCGAGA	HE860456	12.5	17.1	12.9	24.4	4.8	30	3.9	4.3	4.9	8.3	17.4	29.6	4.5	0	10
	GGAATGCTGTCTGGTTCGAGAC	HE863109	5	6.1	8.2	2.7	4.8	17.1	0	1.1	3.3	4.2	4.3	0	0	0	2.9
	GGACCAGGCTTCATTCCC	HE860457	1.3	0	1.2	0	0	0	0	0	0	0	0	0	0	0	0
	GGACCAGGCTTCATTCCCC	HE863111	146.7	154.1	143	184.3	136.3	148.4	155	161.6	134.5	189	191	147.9	171.9	174.7	193.8
	TCGGACCAGGCTTCATTC	HE860457	58.9	64.8	65.6	43.4	28.5	31.4	42.6	54.9	39.4	24.9	28.2	29.6	47.2	54.5	40.2
	TCGGACCAGGCTTCATTCC	HE863113	440.2	408.6	385.7	417.4	261.5	412.4	384.9	339.3	332.9	180.7	256.2	283.9	159.5	280.4	254.1
	TCGGACCAGGCTTCATTCCC	HE860458	706.1	675.3	720.9	441.8	321.8	449.5	586.5	627	546.1	388.4	525.4	387.4	410	512.8	541.2
	TCGGACCAGGCTTCATTCCCC	HE860285	282744.1	279717.9	296598.2	273500	243934.6	239360.7	306036.6	275122.8	248117.2	282794.4	317557.2	199637.5	217491.6	348033.3	350352.6
	TCGGACCAGGCTTCATTCCCCC	HE863115	209.5	212.9	233.3	384.9	160.1	276.9	170.5	137.9	165.6	272.1	191	168.6	174.1	282	236.9
	TCTCGGACCAGGCTTCATTCC	HE860458	110.4	179.8	155.9	273.7	190.2	332.5	11.6	8.6	14.8	24.9	99.9	11.8	28.1	57.7	45.9
	TCTCGGACCAGGCTTCATTCCC	HE863117	0	1.2	1.2	5.4	0	5.7	0	0	0	0	2.2	0	0	1.6	1.4
	TCTCGGACCAGGCTTCATTCCCC	HE860459	2.5	11	9.4	8.1	4.8	12.8	9	4.3	8.2	6.2	4.3	5.9	4.5	9.6	10
	TCTCGGACCAGGCTTCATTCCCCC	HE863119	1.3	0	0	0	0	0	0	0	0	0	0	0	0	0	0
1_32	ATTGACAGAAGAGAGTGAGCAC	HE860459	1.3	0	0	0	0	0	1.3	0	0	0	0	0	0	0	0
	GACAGAAGAGAGTGAGCAC	HE863121	1.3	1.2	0	0	0	0	0	0	0	0	0	0	0	0	0
	GCTCATGTCTCTTTCTGTCAGC	HE860460	5	2.4	7	2.7	4.8	4.3	0	1.1	0	2.1	0	0	1.1	1.6	1.4
	GCTCATGTCTCTTTCTGTCAGCT	HE863123	2.5	0	0	0	0	1.4	0	0	0	0	0	0	0	0	0
	TGACAGAAGAGAGTGAGCA	HE860460	1.3	0	1.2	0	0	0	0	0	0	0	0	0	0	0	0
	TGACAGAAGAGAGTGAGCAC	HE860311	71.5	80.7	97.3	181.6	38	44.2	29.7	53.9	31.2	49.8	17.4	11.8	11.2	11.2	20.1
	TGACAGAAGAGAGTGAGCACA	HE863125	2.5	3.7	0	0	1.6	0	0	0	1.6	2.1	0	0	0	0	0
	TGCTCATGTCTCTTTCTGTCAGC	HE860461	0	2.4	2.3	8.1	1.6	0	1.3	0	0	2.1	0	0	0	1.6	0
	TTGACAGAAGAGAGTGAGCAC	HE863127	8.8	14.7	15.2	21.7	3.2	7.1	2.6	0	1.6	10.4	0	0	0	0	0
1_44	AGGTGGTCAGCATGTCAAACT	HE860461	3.8	2.4	3.5	2.7	0	0	5.2	9.7	0	0	0	3	2.2	3.2	5.7
	TGGCATTCTGTCCACCTCC	HE860451	1.3	0	0	0	0	1.4	0	0	0	0	2.2	0	0	0	0
	TTGGCATTCTGTCCACCT	HE863087	6.3	6.1	7	13.6	11.1	7.1	2.6	1.1	1.6	2.1	0	5.9	0	0	0
	TTGGCATTCTGTCCACCTC	HE860451	18.8	24.5	15.2	13.6	28.5	27.1	1.3	5.4	8.2	2.1	4.3	0	0	1.6	8.6
	TTGGCATTCTGTCCACCTCC	HE860307	89.1	121.1	89.1	127.4	141.1	225.5	80.1	53.9	59	27	26.1	20.7	20.2	35.3	25.8
	TTTGGCATTCTGTCCACCTCC	HE863129	1.3	0	1.2	0	0	0	1.3	1.1	1.6	2.1	0	0	1.1	0	0
1_5	TACAATGAAATCACGGCC	HE860462	0	0	0	0	0	0	0	0	0	2.1	0	0	0	0	0
	TATAAAGAGATGTACTGGACC	HE863131	3.8	2.4	2.3	2.7	0	0	2.6	2.2	0	2.1	4.3	0	2.2	1.6	1.4
	TTATACAATGAAATCACGG	HE860462	0	0	0	0	0	0	0	0	0	0	0	0	1.1	0	0
	TTATACAATGAAATCACGGC	HE860288	8.8	23.2	27	5.4	7.9	7.1	14.2	15.1	3.3	16.6	23.9	20.7	20.2	17.6	18.7
	TTATACAATGAAATCACGGCC	HE860287	125.4	174.9	219.2	70.5	130	81.3	107.2	131.4	147.6	280.4	230.1	378.5	179.7	126.6	277.1
	TTATACAATGAAATCACGGCCG	HE860286	129.2	116.2	148.9	29.8	71.3	37.1	63.3	75.4	62.3	170.3	141.1	195.2	85.4	83.3	208.2
10_1	ACAGGGAACAGGTAGAGCA	HE863133	2.5	1.2	2.3	0	0	0	0	1.1	0	0	0	0	1.1	1.6	0
	ACAGGGAACAGGTAGAGCATG	HE860463	2.5	3.7	8.2	21.7	0	2.9	1.3	2.2	0	0	0	0	0	4.8	0
	ATGCACTGCCTCTTCCCTGGC	HE863135	2.5	3.7	4.7	2.7	1.6	5.7	1.3	0	0	0	0	0	2.2	8	1.4
	TGCACTGCCTCTTCCCTG	HE860463	11.3	3.7	7	5.4	0	8.6	3.9	3.2	0	0	0	3	9	4.8	5.7
	TGCACTGCCTCTTCCCTGG	HE863137	26.3	22	36.3	16.3	1.6	8.6	7.8	10.8	3.3	2.1	0	3	6.7	24	11.5
	TGCACTGCCTCTTCCCTGGC	HE860464	47.7	35.5	32.8	146.4	14.3	65.6	11.6	12.9	8.2	2.1	8.7	8.9	15.7	16	15.8
	TGCACTGCCTCTTCCCTGGCT	HE860428	154.3	137	137.2	168	53.9	119.9	95.6	72.2	90.2	16.6	19.5	14.8	83.1	81.7	64.6
	TGCACTGCCTCTTCCCTGGCTG	HE863139	2.5	6.1	3.5	10.8	1.6	5.7	1.3	2.2	1.6	0	2.2	0	2.2	1.6	4.3
2_31	CCAAAGGGATCGCATTGATCT	HE860464	0	0	0	2.7	1.6	0	0	0	0	0	0	0	0	0	0
	TCCAAAGGGATCGCATTGA	HE863141	0	1.2	1.2	0	0	0	0	0	0	2.1	0	0	0	0	0
	TCCAAAGGGATCGCATTGAT	HE860465	0	1.2	0	0	0	1.4	1.3	1.1	0	0	0	0	0	0	0
	TCCAAAGGGATCGCATTGATC	HE860334	12.5	22	18.8	46.1	44.4	44.2	37.5	43.1	16.4	22.8	23.9	41.4	1.1	3.2	7.2
	TCCAAAGGGATCGCATTGATCT	HE863143	3.8	12.2	15.2	19	20.6	11.4	11.6	19.4	8.2	8.3	4.3	3	1.1	0	2.9
	TCCAAAGGGATCGCATTGATCTA	HE860465	0	0	0	5.4	1.6	4.3	0	0	0	0	0	0	0	0	0
	TCGATGCGATCCCTTGGGA	HE863145	1.3	0	0	0	0	0	0	0	0	0	0	0	0	0	0
	TCGATGCGATCCCTTGGGAAG	HE860337	1.3	4.9	3.5	67.8	30.1	55.7	1.3	4.3	1.6	12.5	6.5	3	2.2	1.6	5.7
	TCGATGCGATCCCTTGGGAAGT	HE860466	0	0	0	2.7	1.6	0	0	0	0	0	0	0	0	0	0
	TGATATTGGATCGATGCGATC	HE863147	0	0	0	0	0	0	1.3	0	0	0	0	0	0	0	0
3_16	ATTGTAGGAATGGGCTGTTTG	HE860466	2.5	0	1.2	0	0	0	1.3	0	0	0	0	0	0	0	0
	CCCAAGCCCGCCCATTCC	HE863149	0	0	0	0	0	0	2.6	0	0	0	0	0	0	0	0
	CCCAAGCCCGCCCATTCCA	HE860467	0	0	0	0	0	0	1.3	1.1	0	0	0	0	0	0	0
	CTTCCCAAGCCCGCCCATTCCA	HE863151	0	0	0	0	0	0	1.3	0	0	0	0	0	0	0	0
	GGAATGGGCTGTTTGGGA	HE860467	13.8	7.3	17.6	16.3	28.5	12.8	3.9	7.5	11.5	10.4	17.4	20.7	11.2	14.4	10
	GGAATGGGCTGTTTGGGAT	HE863153	5	1.2	4.7	0	1.6	2.9	1.3	2.2	0	6.2	4.3	0	2.2	1.6	5.7
	GGAATGGGCTGTTTGGGATG	HE860468	70.2	56.3	92.6	168	68.2	92.8	10.3	24.8	23	22.8	17.4	20.7	69.6	56.1	61.7
	GGAATGGGCTGTTTGGGATGA	HE860347	100.3	84.4	150.1	311.7	111	177	22	49.6	50.8	47.8	34.7	53.2	197.7	86.5	104.8
	GGAATGGGCTGTTTGGGATGAA	HE863155	0	0	0	2.7	0	1.4	0	0	0	0	0	0	1.1	0	0
	GGAATGGGCTGTTTGGGATGAAAG	HE860468	0	0	0	2.7	0	0	0	0	0	0	0	0	2.2	0	1.4
	TAGGAATGGGCTGTTTGGGA	HE863157	2.5	1.2	3.5	0	1.6	0	0	0	0	0	0	0	0	0	1.4
	TTCCCAAGCCCGCCCATT	HE860469	0	0	0	0	0	0	0	0	0	2.1	0	0	0	0	0
	TTCCCAAGCCCGCCCATTC	HE863159	1.3	0	0	0	0	0	0	0	0	0	0	0	0	0	0
	TTCCCAAGCCCGCCCATTCC	HE860469	0	3.7	0	10.8	0	7.1	6.5	4.3	4.9	6.2	8.7	3	0	0	1.4
	TTCCCAAGCCCGCCCATTCCA	HE863161	0	0	2.3	0	1.6	1.4	0	0	1.6	2.1	0	0	0	0	0
	TTCCCAAGCCCGCCCATTCCAA	HE860348	112.9	130.9	99.6	216.8	123.6	182.7	217	225.2	288.7	278.3	212.8	115.3	47.2	38.5	63.2
	TTGTAGGAATGGGCTGTTTGGGA	HE860470	0	0	0	0	0	0	0	0	0	2.1	0	0	0	0	0
	TTTCTTTCATCCCAAACAGCC	HE863163	0	0	0	0	0	0	1.3	0	0	0	0	0	0	0	0
3_28	ATGGTGTCATCCCTCCTGTGACC	HE860470	0	0	0	0	0	0	0	0	0	2.1	0	0	0	0	0
	CCAAATTGAGAGAGAGAGAGAGAG	HE863165	1.3	0	0	0	0	0	0	0	0	0	0	0	0	0	0
	CCATCTTCCTGTGACATGAAC	HE860471	0	0	0	2.7	0	1.4	0	0	0	2.1	0	0	0	0	0
	CGCAGGAGAGATGGCACTG	HE863167	0	0	0	0	0	0	0	0	0	2.1	2.2	3	0	0	0
	GGTGTCATCCCTCCTGTGACC	HE860471	0	0	0	0	1.6	0	0	0	0	2.1	2.2	0	0	0	0
	TCCATCTTCCTGTGACATGA	HE863169	0	0	0	2.7	0	0	0	0	1.6	0	2.2	3	0	0	0
	TCGCAGGAGAGATGGCAC	HE860472	2.5	0	0	0	0	0	0	1.1	0	0	4.3	3	0	0	0
	TCGCAGGAGAGATGGCACTG	HE863171	7.5	2.4	2.3	5.4	0	0	5.2	14	9.8	35.3	39.1	47.3	0	0	5.7
	TCGCAGGAGAGATGGCACTGT	HE860472	1.3	0	2.3	8.1	0	10	1.3	5.4	3.3	8.3	15.2	17.7	1.1	0	1.4
	TCGCAGGAGAGATGGCACTGTC	HE860356	15.1	9.8	15.2	51.5	19	38.5	40	74.3	44.3	110.1	165	139	3.4	4.8	7.2
	TCGCAGGAGAGATGGCACTGTCT	HE863173	0	0	0	2.7	0	0	0	0	1.6	0	0	0	0	0	0
	TGGTGTCATCCCTCCTGTGACC	HE860473	0	0	0	8.1	1.6	4.3	0	2.2	4.9	10.4	8.7	29.6	1.1	0	1.4
	TTCCATCTTCCTGTGACATGA	HE863175	0	0	0	2.7	3.2	1.4	2.6	1.1	1.6	2.1	4.3	23.7	0	0	0
	TTCGCAGGAGAGATGGCAC	HE860473	0	0	0	0	0	0	0	0	0	2.1	0	0	0	0	0
	TTCGCAGGAGAGATGGCACTGTC	HE863177	0	0	1.2	0	0	1.4	2.6	1.1	1.6	2.1	2.2	3	0	0	1.4
4_21	CCCTGCAGTACCTTCCTTTACCC	HE860474	0	0	1.2	2.7	0	0	0	0	0	0	0	0	0	0	0
	GGAGCGACCTGGGATCACATG	HE863179	0	1.2	1.2	21.7	1.6	15.7	0	0	0	0	0	0	1.1	0	0
	GTGTTCTCAGGTCGCCCCTG	HE860474	0	0	2.3	0	0	1.4	0	1.1	3.3	0	0	0	2.2	0	0
	TGTGTTCTCAGGTCGCCCC	HE863181	3.8	2.4	3.5	8.1	4.8	4.3	0	0	0	2.1	0	3	0	14.4	7.2
	TGTGTTCTCAGGTCGCCCCT	HE860475	0	1.2	0	2.7	0	0	0	2.2	0	0	0	3	3.4	1.6	8.6
	TGTGTTCTCAGGTCGCCCCTG	HE860366	356.2	210.4	280.2	311.7	313.9	216.9	31	71.1	54.1	60.2	36.9	35.5	540.3	700.2	723.5
5_14	AATGTTGTCTGGCTCGAG	HE863183	0	1.2	3.5	0	0	1.4	0	0	0	0	0	0	1.1	1.6	1.4
	AATGTTGTCTGGCTCGAGG	HE860475	6.3	13.5	7	8.1	0	1.4	6.5	8.6	0	6.2	10.9	0	6.7	28.8	11.5
	AATGTTGTCTGGCTCGAGGCC	HE863185	0	0	0	2.7	0	0	1.3	1.1	1.6	0	4.3	0	1.1	1.6	0
	AATGTTGTCTGGCTCGAGGCCC	HE860476	0	0	0	0	0	0	1.3	1.1	0	0	0	0	0	0	0
	AATGTTGTCTGGCTCGAGGCCCCT	HE863187	0	0	0	0	0	0	0	2.2	3.3	2.1	0	3	0	0	0
	ACCAGGCTTCATTCCCCC	HE863093	1.3	0	0	0	0	0	0	0	0	0	0	0	0	0	0
	ACGTCGGACCAGGCTTCATTC	HE860476	0	0	0	0	0	0	0	0	0	2.1	0	0	0	0	0
	ACGTCGGACCAGGCTTCATTCCCC	HE863189	1.3	0	1.2	0	0	0	1.3	0	1.6	0	0	0	0	1.6	1.4
	ATGTTGTCTGGCTCGAGG	HE860477	1.3	2.4	3.5	0	0	0	0	0	0	0	2.2	0	1.1	1.6	2.9
	ATTTGGTTCTACATTTAGTGAC	HE863191	1.3	0	0	0	0	0	0	0	0	0	0	0	0	0	0
	CGGACCAGGCTTCATTCC	HE863097	0	0	0	0	0	0	0	1.1	0	0	2.2	0	2.2	0	0
	CGGACCAGGCTTCATTCCC	HE860454	1.3	2.4	0	2.7	1.6	0	1.3	5.4	1.6	0	0	3	1.1	0	0
	CGGACCAGGCTTCATTCCCC	HE863099	282.2	208	289.6	336.1	187	182.7	235.1	213.3	203.4	265.8	251.8	174.5	449.3	299.6	328.7
	CGGACCAGGCTTCATTCCCCC	HE860454	1.3	1.2	0	2.7	3.2	1.4	1.3	1.1	0	2.1	4.3	0	0	1.6	0
	CGTCGGACCAGGCTTCATTCC	HE860477	1.3	0	1.2	0	1.6	0	0	2.2	0	0	0	0	0	0	0
	CGTCGGACCAGGCTTCATTCCC	HE863193	0	0	0	0	0	1.4	0	0	0	0	0	0	3.4	0	0
	CGTCGGACCAGGCTTCATTCCCC	HE860478	3.8	2.4	1.2	0	0	0	1.3	0	3.3	0	2.2	0	2.2	1.6	4.3
	GAATGTTGTCTGGCTCGA	HE863195	1.3	1.2	0	0	0	0	1.3	0	1.6	0	0	0	1.1	0	0
	GAATGTTGTCTGGCTCGAGG	HE860478	5	13.5	7	10.8	1.6	2.9	1.3	2.2	0	0	2.2	3	3.4	14.4	5.7
	GAATGTTGTCTGGCTCGAGGC	HE863197	0	1.2	2.3	2.7	0	0	0	2.2	0	0	0	3	0	0	1.4
	GAATGTTGTCTGGCTCGAGGCC	HE860479	0	2.4	0	0	0	1.4	0	0	0	0	0	0	1.1	1.6	0
	GAATGTTGTCTGGCTCGAGGCCCC	HE863199	0	0	0	0	0	0	0	0	3.3	2.1	0	0	0	0	0
	GACCAGGCTTCATTCCCC	HE860456	1.3	0	0	0	0	0	0	0	1.6	0	0	0	0	0	0
	GGAATGTTGTCTGGCTCG	HE860479	38.9	24.5	44.5	16.3	1.6	5.7	9	26.9	18	14.5	21.7	20.7	22.5	49.7	63.2
	GGAATGTTGTCTGGCTCGA	HE863201	10	11	24.6	5.4	4.8	1.4	11.6	16.2	14.8	22.8	8.7	32.5	5.6	8	24.4
	GGAATGTTGTCTGGCTCGAG	HE860480	6.3	3.7	10.6	16.3	4.8	0	6.5	8.6	3.3	8.3	4.3	11.8	4.5	6.4	14.4
	GGAATGTTGTCTGGCTCGAGG	HE863203	165.6	156.6	174.7	73.2	26.9	49.9	74.9	106.7	80.4	58.1	117.2	263.2	175.2	211.5	353.1
	GGAATGTTGTCTGGCTCGAGGC	HE860480	1.3	1.2	1.2	0	0	0	2.6	1.1	1.6	2.1	0	3	0	4.8	0
	GGACCAGGCTTCATTCCC	HE860457	1.3	0	1.2	0	0	0	0	0	0	0	0	0	0	0	0
	GGACCAGGCTTCATTCCCC	HE863111	146.7	154.1	143	184.3	136.3	148.4	155	161.6	134.5	189	191	147.9	171.9	174.7	193.8
	GTCGGACCAGGCTTCATTC	HE863205	0	0	0	0	0	0	1.3	0	0	0	0	0	0	0	0
	GTCGGACCAGGCTTCATTCC	HE860481	0	0	0	0	0	0	0	1.1	0	0	0	0	1.1	0	0
	GTCGGACCAGGCTTCATTCCC	HE863207	64	117.4	86.7	86.7	49.1	109.9	49.1	56	42.6	29.1	28.2	3	97.7	94.5	68.9
	GTCGGACCAGGCTTCATTCCCC	HE860481	21.3	26.9	29.3	29.8	9.5	14.3	31	29.1	23	33.2	30.4	17.7	51.7	62.5	37.3
	GTCGGACCAGGCTTCATTCCCCC	HE863209	0	2.4	7	0	0	1.4	7.8	8.6	0	2.1	4.3	0	4.5	4.8	7.2
	GTTGTCTGGCTCGAGGCC	HE860482	0	0	0	0	0	0	1.3	0	0	0	0	0	0	0	0
	TAAATGTAGAACCAAATGATCT	HE863211	1.3	0	0	0	0	0	0	0	0	0	0	0	0	0	0
	TCACTAAATGTAGAACCAAATG	HE860482	0	0	0	0	0	0	0	0	0	0	0	0	1.1	0	0
	TCGGACCAGGCTTCATTC	HE860457	58.9	64.8	65.6	43.4	28.5	31.4	42.6	54.9	39.4	24.9	28.2	29.6	47.2	54.5	40.2
	TCGGACCAGGCTTCATTCC	HE863113	440.2	408.6	385.7	417.4	261.5	412.4	384.9	339.3	332.9	180.7	256.2	283.9	159.5	280.4	254.1
	TCGGACCAGGCTTCATTCCC	HE860458	706.1	675.3	720.9	441.8	321.8	449.5	586.5	627	546.1	388.4	525.4	387.4	410	512.8	541.2
	TCGGACCAGGCTTCATTCCCC	HE860285	282744.1	279717.9	296598.2	273500	243934.6	239360.7	306036.6	275122.8	248117.2	282794.4	317557.2	199637.5	217491.6	348033.3	350352.6
	TCGGACCAGGCTTCATTCCCCC	HE863115	209.5	212.9	233.3	384.9	160.1	276.9	170.5	137.9	165.6	272.1	191	168.6	174.1	282	236.9
	TGTCTGGCTCGAGGCCCCTA	HE863213	0	0	0	2.7	0	0	0	0	0	0	0	0	0	0	0
5_3	CCCGCCTTGCATCAACTG	HE860483	0	0	2.3	0	0	0	0	1.1	0	6.2	0	3	0	0	0
	CCCGCCTTGCATCAACTGAA	HE863215	0	0	1.2	0	0	2.9	1.3	1.1	3.3	6.2	8.7	5.9	0	1.6	2.9
	CCCGCCTTGCATCAACTGAAT	HE860483	35.1	44	38.7	75.9	30.1	95.6	62	263.9	242.7	255.4	455.9	257.3	20.2	139.4	208.2
	CCGCCTTGCATCAACTGAAT	HE863217	2.5	0	1.2	0	0	0	2.6	0	0	2.1	4.3	0	0	0	0
	CGCTTGGTGCAGGTCGGGA	HE860484	0	0	0	0	0	0	0	1.1	1.6	2.1	0	3	0	0	0
	CGCTTGGTGCAGGTCGGGAA	HE863219	0	0	0	0	0	0	0	1.1	3.3	6.2	2.2	3	1.1	0	1.4
	CGCTTGGTGCAGGTCGGGAAC	HE860484	0	0	0	2.7	0	0	0	0	0	0	2.2	0	0	0	0
	GCTTGGTGCAGGTCGGGAA	HE863221	0	0	0	0	0	0	1.3	0	0	0	0	0	0	0	0
	GGGTCCCGCCTTGCATCAAC	HE860485	0	0	0	0	0	0	0	0	0	4.2	2.2	0	0	0	0
	GGTCCCGCCTTGCATCAACTGAAT	HE863223	0	0	2.3	0	0	0	0	0	0	2.1	2.2	0	0	0	0
	TCGCTTGGTGCAGGTCGGGA	HE860485	11.3	20.8	15.2	48.8	6.3	14.3	27.1	26.9	68.9	78.9	56.4	65.1	5.6	19.2	18.7
	TCGCTTGGTGCAGGTCGGGAA	HE860370	259.6	254.5	184	219.5	136.3	186.9	193.8	339.3	501.9	494.3	579.6	520.5	104.5	208.3	249.8
	TCGCTTGGTGCAGGTCGGGAACT	HE863225	0	0	0	0	1.6	0	3.9	1.1	8.2	10.4	4.3	3	0	1.6	4.3
	TGGGTCCCGCCTTGCATCAAC	HE860486	6.3	9.8	12.9	24.4	14.3	28.5	28.4	24.8	39.4	45.7	52.1	82.8	4.5	12.8	10
	TGGGTCCCGCCTTGCATCAACT	HE863227	0	0	0	2.7	0	0	0	0	1.6	0	0	0	0	0	0
	TGGTGCAGGTCGGGAACTGCT	HE860486	1.3	1.2	1.2	0	0	0	0	0	0	4.2	0	0	0	0	0
	TTGGTCGGTGGGTGCGAAATGGGT	HE863229	0	0	0	0	0	0	0	0	0	2.1	0	0	0	0	0
6_29	AAGCTCAGGAGGGATAGC	HE860487	1.3	0	0	0	0	0	0	0	0	0	0	0	0	0	0
	AAGCTCAGGAGGGATAGCGC	HE863231	0	0	0	2.7	0	1.4	0	1.1	1.6	0	0	0	0	0	0
	AAGCTCAGGAGGGATAGCGCC	HE860388	70.2	64.8	86.7	178.9	218.7	118.4	153.7	149.7	173.8	211.8	254	224.7	141.5	158.6	113.4
	AGCTCAGGAGGGATAGCGCC	HE860487	0	1.2	0	5.4	1.6	0	0	0	0	2.1	0	0	1.1	0	0
	CGCTATCCATCCTGAGTTTC	HE863233	0	1.2	1.2	8.1	19	2.9	0	0	1.6	0	0	0	0	0	0
	CGCTATCCATCCTGAGTTTCA	HE860488	1.3	1.2	4.7	13.6	42.8	27.1	2.6	5.4	1.6	0	2.2	0	1.1	0	0
	TATTGCGCTATCCATCCTGAGTT	HE863235	0	0	0	0	0	0	1.3	0	0	0	0	0	0	0	0
	TCCATCCTGAGTTTCATGGCT	HE860488	1.3	0	1.2	0	0	0	0	0	0	0	0	0	0	0	0
	TTGCGCTATCCATCCTGAG	HE863237	0	0	0	0	0	0	1.3	0	0	0	0	0	0	0	0
6_30	AAGCTGCCAGCATGATCTGAGC	HE860489	0	0	0	0	0	0	0	0	0	0	0	0	1.1	4.8	0
	AGATCATGTGGTAGCTTCATC	HE863239	5	0	0	0	0	0	0	2.2	1.6	0	0	0	1.1	6.4	7.2
	CTAGATCATGTGGTAGCTTCATC	HE860489	1.3	0	0	0	0	0	1.3	0	1.6	0	0	0	1.1	1.6	0
	GAAGCTGCCAGCATGATCTG	HE863241	0	0	0	0	0	0	0	0	0	0	0	0	1.1	0	2.9
	GAAGCTGCCAGCATGATCTGA	HE860490	1.3	0	1.2	0	0	0	0	0	0	0	0	0	1.1	0	2.9
	GATCATGTGGTAGCTTCATC	HE863243	15.1	11	14.1	0	0	0	10.3	8.6	8.2	0	0	0	37.1	51.3	41.6
	GCTAGATCATGTGGTAGCTTCATC	HE860490	0	0	0	0	1.6	0	0	0	0	0	0	0	1.1	0	4.3
	TAGATCATGTGGTAGCTTCATC	HE863245	0	0	0	0	0	0	0	0	0	0	0	0	1.1	0	0
	TGAAGCTGCCAGCATGAT	HE860491	0	0	0	0	0	0	0	0	0	0	0	0	1.1	0	0
	TGAAGCTGCCAGCATGATC	HE863247	76.5	48.9	62.1	2.7	4.8	2.9	25.8	45.2	41	2.1	8.7	0	21.3	25.6	31.6
	TGAAGCTGCCAGCATGATCT	HE860284	170.6	172.5	161.8	19	17.4	30	153.7	153	88.6	10.4	15.2	14.8	35.9	36.9	48.8
	TGAAGCTGCCAGCATGATCTG	HE860399	1434.8	1105.9	1518.1	43.4	174.4	99.9	496	627	321.5	10.4	13	11.8	775.1	796.4	785.2
	TGAAGCTGCCAGCATGATCTGA	HE860400	1056.1	677.7	907.3	59.6	57.1	34.3	511.5	667.9	408.4	16.6	6.5	3	302.2	427.8	447.9
	TGAAGCTGCCAGCATGATCTGAGC	HE860491	2.5	0	0	0	0	0	1.3	2.2	1.6	0	0	0	2.2	1.6	2.9
	TGTTGAAGCTGCCAGCATGATC	HE863249	1.3	0	0	0	0	0	0	0	0	0	0	0	0	0	0
6_4	AAGCTCAGGAGGGATAGC	HE860487	1.3	0	0	0	0	0	0	0	0	0	0	0	0	0	0
	AAGCTCAGGAGGGATAGCGC	HE863231	0	0	0	2.7	0	1.4	0	1.1	1.6	0	0	0	0	0	0
	AAGCTCAGGAGGGATAGCGCC	HE860388	70.2	64.8	86.7	178.9	218.7	118.4	153.7	149.7	173.8	211.8	254	224.7	141.5	158.6	113.4
	AGCTCAGGAGGGATAGCGCC	HE860487	0	1.2	0	5.4	1.6	0	0	0	0	2.1	0	0	1.1	0	0
	CGCTATCTATCCTGAGTTTCA	HE860492	0	0	0	0	0	0	1.3	0	1.6	8.3	4.3	0	0	0	0
6_7	AATTACTACTTTTGAGTGGTTA	HE863251	1.3	0	0	0	0	0	0	0	0	0	0	0	0	0	0
	ATCTTTCCCAATCCACCCA	HE860492	0	0	0	0	0	0	0	0	0	0	0	0	1.1	0	0
	ATCTTTCCCAATCCACCCATGCC	HE863253	10	12.2	3.5	2.7	1.6	1.4	11.6	16.2	11.5	10.4	6.5	3	14.6	33.6	20.1
	CATGGGTAAGTGGGGAAGA	HE860493	0	0	2.3	0	0	1.4	0	0	0	2.1	0	0	0	0	0
	CATGGGTAAGTGGGGAAGATG	HE863255	18.8	15.9	19.9	37.9	23.8	35.7	1.3	4.3	6.6	4.2	0	3	5.6	8	12.9
	CATGGGTAAGTGGGGAAGATGA	HE860493	5	6.1	3.5	2.7	6.3	2.9	2.6	4.3	0	2.1	6.5	0	2.2	1.6	1.4
	CTTTCCCAATCCACCCATGC	HE863257	0	0	0	0	0	0	0	0	0	2.1	0	0	0	0	0
	CTTTCCCAATCCACCCATGCC	HE860494	0	1.2	1.2	2.7	0	0	1.3	1.1	1.6	4.2	0	5.9	1.1	0	0
	TCCCAATCCACCCATGCC	HE863259	0	0	0	0	0	0	0	0	1.6	2.1	0	0	0	0	0
	TCTTTCCCAATCCACCCA	HE860494	3.8	4.9	3.5	2.7	3.2	1.4	0	1.1	9.8	8.3	0	8.9	0	11.2	7.2
	TCTTTCCCAATCCACCCAT	HE863261	1.3	0	0	0	0	0	0	1.1	0	0	0	0	1.1	0	0
	TCTTTCCCAATCCACCCATG	HE860495	1.3	0	0	0	0	0	2.6	0	0	2.1	0	0	1.1	0	0
	TCTTTCCCAATCCACCCATGC	HE863263	2.5	1.2	3.5	0	1.6	0	1.3	2.2	1.6	6.2	2.2	0	1.1	4.8	1.4
	TCTTTCCCAATCCACCCATGCC	HE860389	652.2	652	726.8	379.4	271.1	298.3	746.6	554.8	657.7	830.7	640.4	505.7	497.6	645.7	551.2
	TCTTTCCCAATCCACCCATGCCT	HE860495	5	0	0	0	0	0	2.6	0	1.6	0	0	0	1.1	0	1.4
	TGGCATGGGTAAGTGGGGAAGA	HE863265	0	0	0	0	0	0	0	0	0	2.1	0	0	1.1	0	0
	TTAGGTTTCCTCTTATTCATCC	HE860496	16.3	39.1	23.4	2.7	4.8	2.9	0	1.1	1.6	8.3	4.3	17.7	10.1	16	10
	TTCCCAATCCACCCATGCCT	HE863267	2.5	0	0	0	0	1.4	1.3	1.1	0	0	0	0	0	0	0
	TTCCCAATCCACCCATGCCTT	HE860496	0	1.2	1.2	0	1.6	0	0	1.1	4.9	0	2.2	0	1.1	1.6	2.9
	TTTCCCAATCCACCCATGCCT	HE863269	0	0	0	2.7	0	0	1.3	1.1	1.6	2.1	4.3	0	0	1.6	0
	TTTCCCAATCCACCCATGCCTT	HE860497	3.8	3.7	3.5	2.7	4.8	1.4	0	3.2	3.3	10.4	4.3	0	4.5	3.2	4.3
	TTTCCCAATCCACCCATGCCTTA	HE863271	0	1.2	1.2	0	4.8	1.4	1.3	2.2	1.6	0	2.2	3	0	4.8	1.4
	TTTCCTCTTATTCATCCCTCT	HE860497	0	0	0	0	0	0	1.3	0	0	0	0	0	0	0	0
7_23	AAGAAAGCTGTGGGAGAACAT	HE863273	0	0	0	2.7	0	0	0	0	0	0	0	0	0	0	0
	AAGAAAGCTGTGGGAGAACATGGC	HE860498	0	1.2	0	0	0	0	1.3	0	0	2.1	0	0	1.1	0	0
	CACAGCTTTCTTGAACTT	HE863275	1.3	3.7	1.2	2.7	3.2	1.4	0	0	0	0	0	0	0	0	1.4
	CCACAGCTTTCTTGAACT	HE860498	0	0	0	2.7	0	1.4	0	0	0	0	0	0	0	0	0
	CTCAAGAAAGCTGTGGGAGA	HE863277	2.5	1.2	4.7	2.7	0	4.3	0	0	0	2.1	2.2	0	1.1	4.8	4.3
	GCTCAAGAAAGCTGTGGGAGA	HE860499	6.3	18.4	10.6	8.1	6.3	14.3	1.3	1.1	1.6	6.2	2.2	8.9	5.6	8	5.7
	TATAAACAAGTCCTGGTCATGCTT	HE863279	0	0	0	2.7	0	0	0	0	0	0	0	0	0	0	0
	TCCACAGCTTTCTTGAACT	HE860499	1.3	0	0	2.7	0	0	0	1.1	0	0	2.2	0	0	0	1.4
	TCCACAGCTTTCTTGAACTT	HE863281	3.8	3.7	4.7	5.4	4.8	4.3	1.3	0	3.3	0	0	3	3.4	0	4.3
	TTCCACAGCTTTCTTGAA	HE860500	0	0	0	2.7	0	0	0	1.1	0	0	0	0	0	0	0
	TTCCACAGCTTTCTTGAAC	HE863283	10	15.9	12.9	27.1	46	37.1	2.6	4.3	4.9	2.1	0	5.9	4.5	4.8	5.7
	TTCCACAGCTTTCTTGAACT	HE860500	76.5	132.1	87.9	146.4	271.1	289.7	16.8	20.5	11.5	8.3	4.3	23.7	40.4	35.3	51.7
	TTCCACAGCTTTCTTGAACTT	HE860413	1138.8	1645.4	1659.9	1658.7	2919.8	2253.4	198.9	377.1	216.5	211.8	149.8	165.6	949.2	741.9	808.2
	TTCCACAGCTTTCTTGAACTTC	HE863285	2.5	0	2.3	5.4	0	4.3	0	1.1	0	0	0	0	3.4	0	1.4
7_24	ACACTGTGGCTCGTTGTGTTGTCA	HE860501	0	0	0	0	0	0	0	0	0	0	0	0	1.1	0	0
	ACGTTATGTTGTCAAATTGTC	HE863287	0	0	0	0	0	0	1.3	1.1	0	0	0	0	0	0	0
	ATGTTGTCAAATTGTCAATC	HE860501	0	0	0	0	1.6	1.4	1.3	0	1.6	0	0	5.9	0	0	0
	CAACGTGACAACACAACGAGC	HE863289	1.3	0	1.2	0	0	2.9	0	0	1.6	0	0	0	0	0	0
	CAACGTGACAACACAACGAGCC	HE860502	10	15.9	12.9	8.1	7.9	12.8	2.6	5.4	6.6	8.3	0	0	0	1.6	2.9
	CACGTTATGTTGTCAAATTGTC	HE863291	0	0	0	2.7	0	1.4	0	0	0	0	0	0	0	0	0
	TATGTTGTCAAATTGTCAAT	HE860502	0	0	0	0	0	0	1.3	0	0	0	0	0	0	0	0
	TATGTTGTCAAATTGTCAATC	HE860414	16.3	9.8	24.6	24.4	12.7	20	24.5	21.5	29.5	10.4	17.4	14.8	12.4	4.8	5.7
	TGAACACAAAGATACATGCCCG	HE863293	0	0	0	0	0	0	1.3	0	0	0	0	0	0	0	0
	TTGACAACGTGACAACACAAC	HE860503	0	1.2	1.2	0	0	0	5.2	2.2	1.6	0	4.3	3	0	0	0
7_25	GACAGAAGAGAGTGAGCAC	HE863121	1.3	1.2	0	0	0	0	0	0	0	0	0	0	0	0	0
	GCTCACTTCTCTCTCTGTCAGC	HE863295	0	0	0	0	0	0	0	0	0	2.1	0	0	0	0	0
	TGACAGAAGAGAGTGAGCA	HE860460	1.3	0	1.2	0	0	0	0	0	0	0	0	0	0	0	0
	TGACAGAAGAGAGTGAGCAC	HE860311	71.5	80.7	97.3	181.6	38	44.2	29.7	53.9	31.2	49.8	17.4	11.8	11.2	11.2	20.1
	TGACAGAAGAGAGTGAGCACA	HE863125	2.5	3.7	0	0	1.6	0	0	0	1.6	2.1	0	0	0	0	0
8_16	CCGACAAGCGTGCTCTCTCTCGTT	HE860503	1.3	0	0	0	0	0	0	0	0	0	0	0	0	0	0
	GTGCTCTCTCTTGTTGTCATG	HE863297	1.3	3.7	4.7	13.6	4.8	0	1.3	0	3.3	2.1	6.5	0	2.2	6.4	5.7
	TGACAACGAGAGAGAGCAC	HE860504	2.5	0	0	0	0	0	0	0	0	0	0	0	0	0	0
	TGACAACGAGAGAGAGCACG	HE863299	0	0	0	2.7	0	1.4	0	0	3.3	0	0	0	0	0	0
	TGACAACGAGAGAGAGCACGC	HE860423	75.3	35.5	21.1	29.8	23.8	41.4	20.7	45.2	32.8	39.5	69.5	62.1	5.6	14.4	8.6
	TTGACAACGAGAGAGAGCAC	HE860504	2.5	1.2	1.2	0	0	1.4	0	0	1.6	0	2.2	0	0	0	1.4
	TTGACAACGAGAGAGAGCACG	HE863301	6.3	0	0	8.1	4.8	2.9	3.9	6.5	3.3	2.1	10.9	0	1.1	1.6	0
	TTGACAACGAGAGAGAGCACGC	HE860505	0	0	0	0	0	0	0	0	0	0	0	0	1.1	0	0
	TTGTCGGCACCCATGAAAGGGCCA	HE863303	0	0	0	2.7	0	0	0	0	0	0	0	0	0	0	0
	TTTGACAACGAGAGAGAGCAC	HE860505	2.5	0	1.2	2.7	0	4.3	1.3	3.2	1.6	4.2	6.5	3	1.1	1.6	0
8_19	AATGTCGTCTGGTTCGAGA	HE863305	0	0	1.2	2.7	3.2	1.4	0	0	0	0	0	0	0	1.6	0
	AATGTCGTCTGGTTCGAGATC	HE860506	1.3	0	0	0	0	1.4	0	0	0	0	0	0	0	0	0
	ATTTCGGACCAGGCTTCATTC	HE863307	3.8	3.7	3.5	2.7	1.6	4.3	1.3	0	0	0	0	3	0	0	0
	ATTTCGGACCAGGCTTCATTCCCC	HE860506	0	1.2	0	2.7	0	0	0	0	0	0	2.2	0	0	0	0
	CGGACCAGGCTTCATTCC	HE863097	0	0	0	0	0	0	0	1.1	0	0	2.2	0	2.2	0	0
	CGGACCAGGCTTCATTCCC	HE860454	1.3	2.4	0	2.7	1.6	0	1.3	5.4	1.6	0	0	3	1.1	0	0
	CGGACCAGGCTTCATTCCCC	HE863099	282.2	208	289.6	336.1	187	182.7	235.1	213.3	203.4	265.8	251.8	174.5	449.3	299.6	328.7
	CGGACCAGGCTTCATTCCCCT	HE863309	2.5	6.1	1.2	8.1	15.9	4.3	0	1.1	4.9	2.1	2.2	0	1.1	3.2	0
	GAATGTCGTCTGGTTCGAGA	HE860507	1.3	7.3	2.3	2.7	0	8.6	0	0	0	0	0	0	0	0	2.9
	GACCAGGCTTCATTCCCC	HE860456	1.3	0	0	0	0	0	0	0	1.6	0	0	0	0	0	0
	GACCAGGCTTCATTCCCCTCA	HE863311	0	0	0	2.7	0	0	0	0	0	0	0	0	0	0	0
	GATTTCGGACCAGGCTTCATTCCC	HE860507	1.3	0	0	2.7	0	0	0	0	0	0	0	0	0	0	0
	GGAATGTCGTCTGGTTCGA	HE863313	1.3	1.2	0	0	1.6	2.9	1.3	1.1	1.6	2.1	0	3	1.1	0	1.4
	GGAATGTCGTCTGGTTCGAGA	HE860508	20.1	15.9	19.9	21.7	28.5	35.7	2.6	5.4	8.2	4.2	0	3	3.4	4.8	5.7
	GGAATGTCGTCTGGTTCGAGAT	HE863315	0	0	0	2.7	0	0	0	0	0	0	0	0	0	0	0
	GGACCAGGCTTCATTCCC	HE860457	1.3	0	1.2	0	0	0	0	0	0	0	0	0	0	0	0
	GGACCAGGCTTCATTCCCC	HE863111	146.7	154.1	143	184.3	136.3	148.4	155	161.6	134.5	189	191	147.9	171.9	174.7	193.8
	GGGAATGTCGTCTGGTTCGAG	HE860508	1.3	0	0	0	0	0	0	0	0	0	0	0	0	0	0
	TCGGACCAGGCTTCATTC	HE860457	58.9	64.8	65.6	43.4	28.5	31.4	42.6	54.9	39.4	24.9	28.2	29.6	47.2	54.5	40.2
	TCGGACCAGGCTTCATTCC	HE863113	440.2	408.6	385.7	417.4	261.5	412.4	384.9	339.3	332.9	180.7	256.2	283.9	159.5	280.4	254.1
	TCGGACCAGGCTTCATTCCC	HE860458	706.1	675.3	720.9	441.8	321.8	449.5	586.5	627	546.1	388.4	525.4	387.4	410	512.8	541.2
	TCGGACCAGGCTTCATTCCCC	HE860285	282744.1	279717.9	296598.2	273500	243934.6	239360.7	306036.6	275122.8	248117.2	282794.4	317557.2	199637.5	217491.6	348033.3	350352.6
	TCGGACCAGGCTTCATTCCCCT	HE863317	37.6	41.6	39.9	27.1	12.7	21.4	59.4	47.4	41	31.2	28.2	35.5	33.7	32	40.2
	TCGGACCAGGCTTCATTCCCCTC	HE860509	0	0	1.2	0	0	0	0	0	0	2.1	0	0	0	0	0
	TTCGGACCAGGCTTCATTCC	HE863319	1.3	0	2.3	0	4.8	1.4	0	1.1	0	0	0	0	0	0	0
	TTCGGACCAGGCTTCATTCCC	HE860509	184.4	223.9	195.8	273.7	416.9	299.7	49.1	62.5	47.6	24.9	17.4	23.7	35.9	49.7	43.1
	TTCGGACCAGGCTTCATTCCCC	HE863321	153	168.8	158.3	219.5	280.6	191.2	117.5	101.3	95.1	27	32.6	20.7	35.9	57.7	44.5
	TTCGGACCAGGCTTCATTCCCCT	HE860510	1.3	0	0	0	1.6	0	0	0	0	0	0	0	0	0	0
	TTGAGGGGAATGTCGTCTGG	HE863323	1.3	0	0	0	0	0	0	0	0	0	0	0	0	0	0
	TTTCGGACCAGGCTTCATTCC	HE860510	67.7	104	85.6	103	187	114.2	41.3	25.9	24.6	4.2	6.5	20.7	14.6	11.2	15.8
8_21	CACGTGCTCCCCTTCTCC	HE863325	0	0	0	0	0	0	0	0	0	2.1	0	0	0	0	0
	CACGTGCTCCCCTTCTCCAAC	HE860511	2.5	1.2	2.3	0	4.8	10	10.3	4.3	11.5	12.5	17.4	20.7	3.4	1.6	4.3
	TGGAGAAGCAGGGCACGTGCA	HE860424	55.2	30.6	24.6	62.3	7.9	45.7	14.2	28	21.3	20.8	17.4	29.6	6.7	8	4.3

*BF, pink; F, bloom; GF, swollen flower bud; O, half-inch green; GL, swollen leaf bud*.

### IsomiRs identification and analysis

IsomiRs at each locus were blasted against miRBase. In some cases no mismatches were reported with the conserved sequences present in miRBase (e.g., miR403, miR394, miR166, miR156) while in some others mismatches were present and related to differences in the sequence and/or in its length. Detailed blast results are reported in File S8 in Supplementary Material which reports blast results based both on mature sequences (sheet “mature”) and precursor sequences (sheet “precursors”) deposited in miRBase. The file reports the matching sequence with the lowest *e*-value. When more than one matching sequence, belonging to different miRNA families, were found to have the same *e*-value all of them were reported.

Some miRNA families have more than one putative locus, therefore miRDeeP assigned common reads to all the possible loci. Both miRNA and miRNA*-related reads were identified at each locus. In some cases putative miRNAs* were identified on the basis of the alignment orientation (± with miRNA mature sequence deposited in miRBase) in some others the miRNA* sequences were already deposited in miRBase. The results of Table 2 highlight that some loci are characterized by a larger set of variants than others.

In the majority of the loci the most frequent read for a specific locus was the same in all the tested samples and across all the replicates of a sample (Table 2). Only in a few cases were some differences detected among samples or among replicates belonging to the same sample. Locus named 3_16 is particularly interesting because all the replicates of sample O have as the most frequent read the one corresponding to miRNA* (Table 2).

In some loci also the second most frequent read referred to the mature miRNA was the same in all the replicates of a sample and in all the samples. The second most frequent read was often obtained by a different cutting site at 5′ or 3′ ends. As reported above, miRNA*-related reads have also been identified by miRDeep-P for most of the 26 loci and length variability was detected for both 5′ and 3′ends.

Target analysis was carried out by psRNATarget. The whole set of targets identified is reported in File S9 in Supplementary Material.

### Intra- and inter-samples analysis

**Figure 1:**
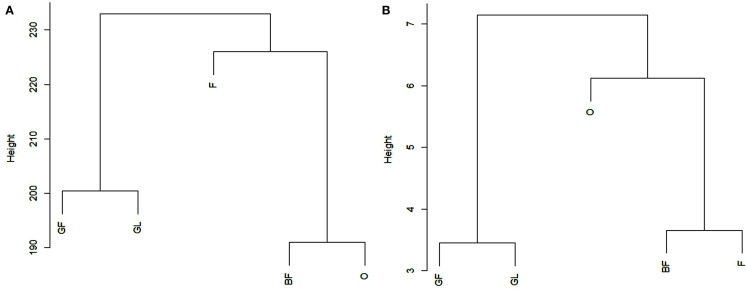
**Reports the results obtained by cluster analysis of the five tissues on the basis of the frequencies of all the reads (A) or on the basis of the frequency of the most frequent read in each locus (B)**. For each tissue, the average frequency across the three replicates was considered (miRNA* -related reads are included). In **(B)**, the analysis included only those loci where the most frequent read was the same in all the samples. BF, pink; F, bloom; GF, swollen flower bud; O, half-inch green; GL, swollen leaf bud.

**Figure A1:**
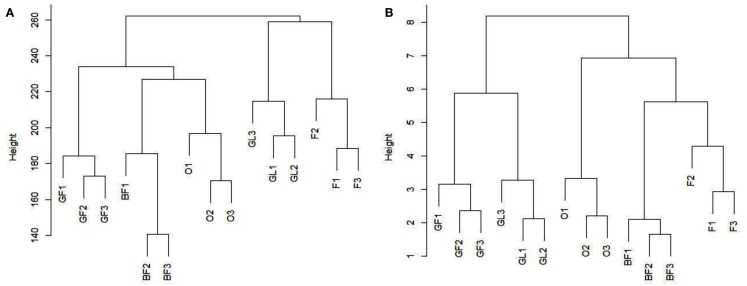
**Reports the results obtained by cluster analysis as in Figure 1 but in this case without averaging the replicates of each sample**. In details, **(A)** reports the results obtained by cluster analysis of the five tissues on the basis of the frequencies of all the reads, while **(B)** reports results obtained on the basis of the frequency of the most frequent read in each locus. Abbreviations: pink = BF, bloom = F, swollen flower bud = GF, half-inch green = O, swollen leaf bud = GL.

The average Pearson correlation between all the possible pairs of replicates belonging to the same biological sample was calculated, in order to evaluate whether it was in agreement with the “Standards, guidelines, and best practices for RNA-seq” adopted by ENCODE Consortium.^4^ Average correlation coefficients were equal to 0.98 for BF, 0.95 for F, 0.98 for GF, 0.95 for GL, and 0.97 for O. For the sake of completeness and in order to allow a comparison between related and unrelated samples, we also calculated the average Pearson correlation between samples of different tissues, which was equal to 0.66 on the basis of the reads reported in Table 2. All the Pearson coefficients are reported in File S10 in Supplementary Material. Figure 1A reports the results obtained from clustering the five samples on the basis of all the reads frequencies (average frequencies of three replicates, reads included miRNA*-related reads; reads assigned by miRDeep-P to more than one locus were counted once) at the 26 loci analyzed. Additionally, a clustering analysis was performed by considering only the count of the most frequent read in each locus. The analysis included those loci where the most frequent read was the same in all the samples (16 different reads, Figure 1B). Figure A1 in Appendix reports clustering results obtained without averaging the three replicates of each sample. As it can be seen, replicates are always grouped correctly.

A *t*-test was also performed for all the possible comparisons of biological samples (File S11 in Supplementary Material). The most frequent isomiR (highlighted in yellow in File S11 in Supplementary Material) is frequently the one able to distinguish the higher number of samples (e.g., locus 4_21, locus 6_4). Some miRNA-related reads are able to differentiate most of the analyzed samples: e.g., miR398 and miR167 got 8 significant comparisons out of 10.

## Discussion

To assess the putative biological significance of isomiRs in peach, in the present study we carried out miRNAs profiling by sequencing three replicates of five biological samples arising from a set of different organs and/or phenological stages. Actually, variants of miRNAs are commonly found in deep sequencing experiments but their functional meaning and stability is still under investigation in plants.

Twenty-six miRNA putative loci expressed in all samples analyzed have been identified by miRdeep-P and analyzed for miRNA population heterogeneity. The average length of miRNA associated reads was included between 18 nt and 24 nt. Several previous works reported a miRNA length in plants included between 22 nt and 24 nt. The identification of miRNA* associated reads provides more evidence about reliability of the loci identified by miRDeep-P.

All the analyzed loci show miRNA length variants but tend to maintain the uridine at the 5′ end, in those cases where uridine is the first base of the most abundant isomiR. As reported above, uridine is the most frequent nucleotide in AGO1 association, perhaps explaining the drive to maintain it at the 5′ end. Ebhardt et al. (2009) reported examples of miRNA with 5′ deletions and 3′ uridine additions that create a different distribution in AGO complexes. As an example, ath-miR822 was determined to reside almost exclusively in the AGO1 complex while its modified variant with a U deletion at 5′ end and a UU addition at 3′end was found equally in AGO1 and AGO4 complexes.

The difference in read count between the first most frequent read and the second most frequent read varies among loci being in some cases minimal (e.g., locus 1_5) while in some others it is quite consistent (e.g., loci 4_21, 6_4). In some loci the second most frequent read was the same in all the replicates of a sample and in all the different samples. The presence of the same isomiRs in different biological replicates of a sample and in different tissues demonstrate that the generation of most of the detected isomiRs is not random. The importance of evaluating the correlation between biological replicates from RNA-seq experiments has been discussed previously in several papers (Oshlack et al., 2010; Hansen et al., 2011). As above reported, the correlation among biological replicates has been calculated to check the reliability of the experiment on the basis of the “Standards, guidelines, and best practices for RNA-seq” adopted by ENCODE Consortium which requires that the Pearson correlation of gene expression between two biological replicates for RNAs that are detected in both samples using RPKM or read counts should be between 0.92 and 0.98. Regarding the present work, the average Pearson correlation between all the possible pairs of replicates belonging to the same biological sample was greater than or equal to 0.95 for all the tested samples, in agreement with the required standards. Clustering results and *t*-test reported in Figure 1 and File S11 in Supplementary Material, respectively, show that it is possible to clearly distinguish among samples and to group them in a functional way. However, when considering Figure A1 in Appendix obtained without averaging replicates of each sample, it should be noted that clustering results seem to be more confident when only the most frequent read is taken into account: BF (pink) and F (bloom) are more strictly related being two subsequent phenological stages so it is expected to find a closer relationship between them.

The co-existence of different variants with a similar level of expression could imply a biological role for all of them. Locus 1_26 shows such an example: in this case there are two prevalent isomiRs (HE860305 and HE860450) that differ for one T at the 5′ end. For both the isomiRs there are then variants at the 3′ end with different lengths.

Target analysis carried out by psRNATarget (File S9 in Supplementary Material) revealed that in many cases isomiRs share the same target. However, because AGO invariably catalyzes the cleavage of targets opposite the bond between nucleotides 10 and 11 from the 5′ end of the miRNA, the cleavage products are different when there is a shift toward the 5′ end or nucleotide addition at the 5′ end of the miRNA mature sequence. Differences in cleavage sites among members of the same miRNA family have been recently studied in rice by Jeong et al. (2011) highlighting a different abundance of specific cleavage sites among plant organs.

A very interesting finding is related to the biological role of miRNA*. Despite the general consensus that miRNAs* have no regulatory activity, several recent publications have provided evidence about their biological function (Mah et al., 2010). In our results, isomiRs have been found also for miRNAs*. As an example, at locus 3_16 the conserved miRNA* has a high number of length variants, most due to a variable 3′end. Locus 3_16 codes for miR482: the miRNA* sequence deposited in miRBase was actually the most frequent read (HE860347) in all the three replicates of sample O (half-inch green) with an average ratio miRNA/miRNA* equal to 0.4. GF and GL showed an average ratio of miRNA/miRNA* equal to 5.7, while in BF and F the ratio was close to one in two out of three replicates. Similar results have been previously found in mammals by Kuchenbauer et al. (2012) that classified miRNA/miRNA* ratios into groups showing that about 50% of all miRNA duplexes revealed high ratios (>100) consistent with a strong preferential processing of one dominant miRNA strand. About 24% had intermediate ratios (between 100 and 10), about 13% showed low ratios (between 10 and 1), while another 13% showed inverted ratios (<1). The finding that miRNAs can display tissue-dependent miRNA arm selection opposes the general consensus that only one strand is highly dominant for any given miRNA duplex and opens insights into the possible biological function of selective accumulation of miRNA*. A recent review of Sunkar et al. (2012), discusses several studies showing that miRNA* tend to accumulate at a high level under particular conditions. As an example, miR393* accumulates at a high level during infection of *P. syringae* in *Arabidopsis* leaves and promotes plant resistance to bacterial infection. Mir399* is accumulated at high levels during phosphate deprivation in *Arabidopsis* and miR395* accumulates at high levels in *Sorghum* grown in optimal nutrient conditions.

PsRNATarget has been used to investigate possible target genes for miR482 and miR482* at locus 3_16. MiRNA482 target a peach sequence coding for a “probable receptor-like protein kinase” (expectation = 2, target accessibility = 17.288), while miRNA482* targets a NADH dehydrogenase gene (expectation = 3, target accessibility = 8.463). Examples of different targets for a pair of miRNA/miRNA* are reported in previous studies (Sunkar et al., 2012). Mir393 and miR393* target two entirely different gene families (TIR1 and SNARE) both involved in pathogen resistance of host plant. The possibility that a target-dependent strand selection based on the presence in the cell of miRNA or miRNA* targets might influence the selection of the active miRNA arm has been discussed by other authors. For instance Chatterjee and Grosshans (2009) reported that mRNAs can stabilize their cognate miRNAs thus suggesting coordinated RISC assembly which depends on a miRNA and its target levels.

Results obtained in the present work contribute to a deeper view of the miRNome complexity and to a better exploitation of the mechanism of action of these tiny regulators. The exact definition of the entire repertoire of peach miRNAs is in fact a prerequisite for a correct description of miRNAs whose expression is altered in response to specific developmental conditions or environmental stimuli. Future experiments based on small RNA-seq coupled with RNA-seq on the same samples will be carried out to highlight more clearly the possible biological role of miRNA isomiRs in plants.

## Conflict of Interest Statement

The authors declare that the research was conducted in the absence of any commercial or financial relationships that could be construed as a potential conflict of interest.

## Supplementary Material

The Supplementary Material for this article can be found online at: http://www.frontiersin.org/Plant_Genetics_and_Genomics/10.3389/fpls.2012.00165/abstract

File S1 **Reports the miRNA coding loci identified by miRDeep-P in pink sample**.

File S2 **Reports the miRNA coding loci identified by miRDeep-P in bloom sample**.

File S3 **Reports the miRNA coding loci identified by miRDeep-P in swollen flower bud sample**.

File S4 **Reports the miRNA coding loci identified by miRDeep-P in half-green sample**.

File S5 **Reports the miRNA coding loci identified by miRDeep-P in swollen leaf bud sample**.

File S6 **Reports a summary of the miRNA coding loci identified by miRDeep-P**.

File S7 **Reports the link between locus name and locus position**.

File S8 **Reports the results of the blast analysis against known plant miRNAs**.

File S9 **Reports target analysis for all the identified isomiRs**.

File S10 **Reports Pearson correlation coefficients between all the possible pairs of replicates belonging to the same biological sample, as well as samples from different tissues**.

File S11 **Reports the results of the *t*-test which was performed for all the possible comparisons of biological samples. The most frequent isomiR in each locus is highlighted in yellow**.

## Footnotes

^1^www.mirbase.org

^2^http://srna-tools.cmp.uea.ac.uk/plant/cgi-bin/srna-tools.cgi

^3^http://plantgrn.noble.org/psRNATarget

^4^http://encodeproject.org/ENCODE/protocols/dataStandards/RNA_standards_v1_2011_May.pdf
