# Supplementary material for: A Survey of MicroRNA Length Variants Contributing to miRNome Complexity in Peach (Prunus Persica L.)
Source: Front Plant Sci. 2012 Jul 26;3:165. doi: 10.3389/fpls.2012.00165 (PMC3405489; doi:10.3389/fpls.2012.00165)
Supplement: File S9 — Reports target analysis for all the identified isomiRs. [file 26922_Faccioli_DataSheet9.DOCX]

| **miRNA locus** | **reads** | **Target ID** | **Expectation (E)** | **Target accessibility**  **(UPE)** | **Target description as from PSRNAtarget** |
| --- | --- | --- | --- | --- | --- |
|  |  |  |  |  |  |
| **1_10** | AGTTTGTGCGTGAATCGAACC |  |  |  |  |
|  | CAGTTTGTGCGTGAATCGAAC |  |  |  |  |
|  | TTAGATTCACGCACAAAC |  |  |  |  |
|  | TTAGATTCACGCACAAACT |  |  |  |  |
|  | TTAGATTCACGCACAAACTC | TC9016 | 2.5 | 14.32 | homologue to UniRef100_Q0G7J0 Cluster: HAD-superfamily hydrolase subfamily IA; n=1; Fulvimarina pelagi HTCC2506\|Rep: HAD-superfamily hydrolase subfamily IA - Fulvimarina pelagi HTCC2506, partial (7%) |
|  | TTAGATTCACGCACAAACTCG | TC9016 | 2.5 | 14.32 | homologue to UniRef100_Q0G7J0 Cluster: HAD-superfamily hydrolase subfamily IA; n=1; Fulvimarina pelagi HTCC2506\|Rep: HAD-superfamily hydrolase subfamily IA - Fulvimarina pelagi HTCC2506, partial (7%) |
|  |  |  |  |  |  |
| **1_15** | AACCACAAATCTCTTGGACTCCTG | DW346031 | 1 | 24.659 | similar to UniRef100_Q0J9S9 Cluster: Os04g0633200 protein; n=1; Oryza sativa Japonica Group\|Rep: Os04g0633200 protein - Oryza sativa subsp. japonica (Rice), partial (4%) |
|  | AAGAGATTTGTGGTTACTCAC | DW346105 | 0.5 | 11.836 | UniRef100_A4VTF7 Cluster: Predicted membrane protein; n=2; Streptococcus suis\|Rep: Predicted membrane protein - Streptococcus suis (strain 05ZYH33), partial (6%) |
|  | AAGAGATTTGTGGTTACTCACC | DW346105 | 0.5 | 11.836 | UniRef100_A4VTF7 Cluster: Predicted membrane protein; n=2; Streptococcus suis\|Rep: Predicted membrane protein - Streptococcus suis (strain 05ZYH33), partial (6%) |
|  | AAGAGATTTGTGGTTACTCACCG | DW346105 | 0.5 | 11.836 | UniRef100_A4VTF7 Cluster: Predicted membrane protein; n=2; Streptococcus suis\|Rep: Predicted membrane protein - Streptococcus suis (strain 05ZYH33), partial (6%) |
|  | AAGAGATTTGTGGTTACTCACCGT | DW346105 | 0.5 | 11.836 | UniRef100_A4VTF7 Cluster: Predicted membrane protein; n=2; Streptococcus suis\|Rep: Predicted membrane protein - Streptococcus suis (strain 05ZYH33), partial (6%) |
|  | AGAGATTTGTGGTTACTCAC | DW346105 | 0.5 | 11.838 | UniRef100_A4VTF7 Cluster: Predicted membrane protein; n=2; Streptococcus suis\|Rep: Predicted membrane protein - Streptococcus suis (strain 05ZYH33), partial (6%) |
|  | AGAGATTTGTGGTTACTCACCG | DW346105 | 0.5 | 11.838 | UniRef100_A4VTF7 Cluster: Predicted membrane protein; n=2; Streptococcus suis\|Rep: Predicted membrane protein - Streptococcus suis (strain 05ZYH33), partial (6%) |
|  | AGAGATTTGTGGTTACTCACCGT | DW346105 | 0.5 | 11.838 | UniRef100_A4VTF7 Cluster: Predicted membrane protein; n=2; Streptococcus suis\|Rep: Predicted membrane protein - Streptococcus suis (strain 05ZYH33), partial (6%) |
|  | AGAGATTTGTGGTTACTCACCGTT | DW346105 | 0.5 | 11.838 | UniRef100_A4VTF7 Cluster: Predicted membrane protein; n=2; Streptococcus suis\|Rep: Predicted membrane protein - Streptococcus suis (strain 05ZYH33), partial (6%) |
|  | ATTTACATCCAACGGTGAGTAACC | DW343340 | 0 | 16.992 |  |
|  | CAAGAGATTTGTGGTTACTCA | DW343252 | 0.5 | 16.113 | similar to UniRef100_A9BF29 Cluster: Chromosome segregation and condensation protein ScpA; n=1; Petrotoga mobilis SJ95\|Rep: Chromosome segregation and condensation protein ScpA - Petrotoga mobilis SJ95, partial (10%) |
|  | CAAGAGATTTGTGGTTACTCACC | DW343252 | 0.5 | 16.113 | similar to UniRef100_A9BF29 Cluster: Chromosome segregation and condensation protein ScpA; n=1; Petrotoga mobilis SJ95\|Rep: Chromosome segregation and condensation protein ScpA - Petrotoga mobilis SJ95, partial (10%) |
|  | CAAGAGATTTGTGGTTACTCACCG | DW343252 | 0.5 | 16.113 | similar to UniRef100_A9BF29 Cluster: Chromosome segregation and condensation protein ScpA; n=1; Petrotoga mobilis SJ95\|Rep: Chromosome segregation and condensation protein ScpA - Petrotoga mobilis SJ95, partial (10%) |
|  | CCAAGAGATTTGTGGTTACTCA | DW343252 | 0.5 | 16.176 | similar to UniRef100_A9BF29 Cluster: Chromosome segregation and condensation protein ScpA; n=1; Petrotoga mobilis SJ95\|Rep: Chromosome segregation and condensation protein ScpA - Petrotoga mobilis SJ95, partial (10%) |
|  | TCCAAGAGATTTGTGGTTACTCAC | DW343252 | 0.5 | 16.146 | similar to UniRef100_A9BF29 Cluster: Chromosome segregation and condensation protein ScpA; n=1; Petrotoga mobilis SJ95\|Rep: Chromosome segregation and condensation protein ScpA - Petrotoga mobilis SJ95, partial (10%) |
|  |  |  |  |  |  |
| **1_25** | CGAAACCTCCCATTCCAA |  |  |  |  |
|  | GAGAGGTTGCCGGAAAGA |  |  |  |  |
|  | GGGTGAGAGGTTGCCGGAAA | DW345559 | 2.5 | 6.396 | similar to UniRef100_Q4SMG2 Cluster: Chromosome undetermined SCAF14551, whole genome shotgun sequence; n=1; Tetraodon nigroviridis\|Rep: Chromosome undetermined SCAF14551, whole genome shotgun sequence - Tetraodon nigroviridis (Green puffer), partial (5%) |
|  | GGGTGAGAGGTTGCCGGAAAG | DW345559 | 2.5 | 6.396 | similar to UniRef100_Q4SMG2 Cluster: Chromosome undetermined SCAF14551, whole genome shotgun sequence; n=1; Tetraodon nigroviridis\|Rep: Chromosome undetermined SCAF14551, whole genome shotgun sequence - Tetraodon nigroviridis (Green puffer), partial (5%) |
|  | GGGTGAGAGGTTGCCGGAAAGA | DW345559 | 2.5 | 6.396 | similar to UniRef100_Q4SMG2 Cluster: Chromosome undetermined SCAF14551, whole genome shotgun sequence; n=1; Tetraodon nigroviridis\|Rep: Chromosome undetermined SCAF14551, whole genome shotgun sequence - Tetraodon nigroviridis (Green puffer), partial (5%) |
|  | GGGTGAGAGGTTGCCGGAAAGAA | DW345559 | 2.5 | 6.396 | similar to UniRef100_Q4SMG2 Cluster: Chromosome undetermined SCAF14551, whole genome shotgun sequence; n=1; Tetraodon nigroviridis\|Rep: Chromosome undetermined SCAF14551, whole genome shotgun sequence - Tetraodon nigroviridis (Green puffer), partial (5%) |
|  | GGTGAGAGGTTGCCGGAAAGAAT | DW345559 | 3 | 6.404 | similar to UniRef100_Q4SMG2 Cluster: Chromosome undetermined SCAF14551, whole genome shotgun sequence; n=1; Tetraodon nigroviridis\|Rep: Chromosome undetermined SCAF14551, whole genome shotgun sequence - Tetraodon nigroviridis (Green puffer), partial (5%) |
|  | TCCGAAACCTCCCATTCCAA | DW342241 | 3 | 14.171 | similar to UniRef100_Q9AZI4 Cluster: Orf25; n=2; root\|Rep: Orf25 - Lactococcus phage bIL310, partial (8%) |
|  | TCCGAAACCTCCCATTCCAAT | DW342241 | 3 | 14.171 | similar to UniRef100_Q9AZI4 Cluster: Orf25; n=2; root\|Rep: Orf25 - Lactococcus phage bIL310, partial (8%) |
|  | TCCGAAACCTCCCATTCCAATG | DW342241 | 3 | 14.171 | similar to UniRef100_Q9AZI4 Cluster: Orf25; n=2; root\|Rep: Orf25 - Lactococcus phage bIL310, partial (8%) |
|  | TTCCGAAACCTCCCATTCCAA | DW342241 | 3 | 14.511 | similar to UniRef100_Q9AZI4 Cluster: Orf25; n=2; root\|Rep: Orf25 - Lactococcus phage bIL310, partial (8%) |
|  | TTCCGAAACCTCCCATTCCAAT | DW342241 | 3 | 14.511 | similar to UniRef100_Q9AZI4 Cluster: Orf25; n=2; root\|Rep: Orf25 - Lactococcus phage bIL310, partial (8%) |
|  | TTGGGTGAGAGGTTGCCGGAA | BU041750 | 2.5 | 9.364 | similar to UniRef100_A7PM80 Cluster: Chromosome chr14 scaffold_21, whole genome shotgun sequence; n=1; Vitis vinifera\|Rep: Chromosome chr14 scaffold_21, whole genome shotgun sequence - Vitis vinifera (Grape), partial (31%) |
|  | TTGGGTGAGAGGTTGCCGGAAA | BU041750 | 2.5 | 9.364 | similar to UniRef100_A7PM80 Cluster: Chromosome chr14 scaffold_21, whole genome shotgun sequence; n=1; Vitis vinifera\|Rep: Chromosome chr14 scaffold_21, whole genome shotgun sequence - Vitis vinifera (Grape), partial (31%) |
|  | TTTCCGAAACCTCCCATT |  |  |  |  |
|  | TTTCCGAAACCTCCCATTC |  |  |  |  |
|  | TTTCCGAAACCTCCCATTCC |  |  |  |  |
|  | TTTCCGAAACCTCCCATTCCA |  |  |  |  |
|  | TTTCCGAAACCTCCCATTCCAA |  |  |  |  |
|  | TTTCCGAAACCTCCCATTCCAAT |  |  |  |  |
|  |  |  |  |  |  |
| **1_26** | AAAAAGACTCAACAACCCATGTTT | BU040232 | 3 | 12.887 | similar to UniRef100_O22670 Cluster: Ag13 protein precursor; n=1; Alnus glutinosa\|Rep: Ag13 protein precursor - Alnus glutinosa (Alder), partial (14%) |
|  | AAAAGACTCAACAACCCATGT |  |  |  |  |
|  | AAAAGACTCAACAACCCATGTTT |  |  |  |  |
|  | AAAGACTCAACAACCCATGT |  |  |  |  |
|  | AAAGGCATAGTAGGGTTTAGGA |  |  |  |  |
|  | AAAGGCATAGTAGGGTTTAGGAAG |  |  |  |  |
|  | AAGGCATAGTAGGGTTTAGGAAGT |  |  |  |  |
|  | ACCCCGCCCATTCCAAATATT | TC17136 | 3 | 18.458 | homologue to UniRef100_A7PF18 Cluster: Chromosome chr11 scaffold_13, whole genome shotgun sequence; n=1; Vitis vinifera\|Rep: Chromosome chr11 scaffold_13, whole genome shotgun sequence - Vitis vinifera (Grape), partial (93%) |
|  | ACCCCGCCCATTCCAAATATTT | TC17136 | 3 | 18.458 | homologue to UniRef100_A7PF18 Cluster: Chromosome chr11 scaffold_13, whole genome shotgun sequence; n=1; Vitis vinifera\|Rep: Chromosome chr11 scaffold_13, whole genome shotgun sequence - Vitis vinifera (Grape), partial (93%) |
|  | ATATTTTCTAAGCCTACTGTC | TC11345 | 2 | 11.833 | homologue to UniRef100_Q2QDF4 Cluster: CDPK-related protein kinase; n=1; Gossypium hirsutum\|Rep: CDPK-related protein kinase - Gossypium hirsutum (Upland cotton) (Gossypium mexicanum), partial (39%) |
|  | CAAATATTTTCTAAGCCTACTGTC | TC15592 | 2.5 | 22.26 | similar to UniRef100_A7QF28 Cluster: Chromosome chr16 scaffold_86, whole genome shotgun sequence; n=1; Vitis vinifera\|Rep: Chromosome chr16 scaffold_86, whole genome shotgun sequence - Vitis vinifera (Grape), partial (93%) |
|  | CATAGTAGGGTTTAGGAA |  |  |  |  |
|  | CATAGTAGGGTTTAGGAAGTT | DW341867 | 3 | 17.985 | similar to UniRef100_Q6URA2 Cluster: TIR-NBS-LRR type R protein 7; n=1; Malus baccata\|Rep: TIR-NBS-LRR type R protein 7 - Malus baccata, partial (5%) |
|  | CATAGTAGGGTTTAGGAAGTTT | DW341867 | 3 | 17.985 | similar to UniRef100_Q6URA2 Cluster: TIR-NBS-LRR type R protein 7; n=1; Malus baccata\|Rep: TIR-NBS-LRR type R protein 7 - Malus baccata, partial (5%) |
|  | CATAGTAGGGTTTAGGAAGTTTT | DW341867 | 3 | 17.985 | similar to UniRef100_Q6URA2 Cluster: TIR-NBS-LRR type R protein 7; n=1; Malus baccata\|Rep: TIR-NBS-LRR type R protein 7 - Malus baccata, partial (5%) |
|  | CATAGTAGGGTTTAGGAAGTTTTT | DW341867 | 3 | 17.985 | similar to UniRef100_Q6URA2 Cluster: TIR-NBS-LRR type R protein 7; n=1; Malus baccata\|Rep: TIR-NBS-LRR type R protein 7 - Malus baccata, partial (5%) |
|  | CTTTGCCAACCCCGCCCATTCC | FC862909 | 3 | 23.774 | similar to UniRef100_Q9ZPV5 Cluster: Nucleolar complex protein 2 homolog; n=1; Arabidopsis thaliana\|Rep: Nucleolar complex protein 2 homolog - Arabidopsis thaliana (Mouse-ear cress), partial (26%) |
|  | CTTTGCCAACCCCGCCCATTCCA | FC862909 | 3 | 23.774 | similar to UniRef100_Q9ZPV5 Cluster: Nucleolar complex protein 2 homolog; n=1; Arabidopsis thaliana\|Rep: Nucleolar complex protein 2 homolog - Arabidopsis thaliana (Mouse-ear cress), partial (26%) |
|  | CTTTGCCAACCCCGCCCATTCCAA | FC862909 | 3 | 23.774 | similar to UniRef100_Q9ZPV5 Cluster: Nucleolar complex protein 2 homolog; n=1; Arabidopsis thaliana\|Rep: Nucleolar complex protein 2 homolog - Arabidopsis thaliana (Mouse-ear cress), partial (26%) |
|  | GAAAGGCATAGTAGGGTTTAGGA | AM289902 | 3 | 8.759 | similar to UniRef100_Q67V42 Cluster: MutT domain protein-like; n=1; Oryza sativa Japonica Group\|Rep: MutT domain protein-like - Oryza sativa subsp. japonica (Rice), partial (45%) |
|  | GAAAGGCATAGTAGGGTTTAGGAA | AM289902 | 3 | 8.759 | similar to UniRef100_Q67V42 Cluster: MutT domain protein-like; n=1; Oryza sativa Japonica Group\|Rep: MutT domain protein-like - Oryza sativa subsp. japonica (Rice), partial (45%) |
|  | GCCAACCCCGCCCATTCCAA |  |  |  |  |
|  | GGAATGAGCGTGTTGGAAA |  |  |  |  |
|  | GGAATGAGCGTGTTGGAAAA | TC10229 | 3 | 5.815 | similar to UniRef100_P42738 Cluster: Chorismate mutase, chloroplast precursor; n=2; Arabidopsis thaliana\|Rep: Chorismate mutase, chloroplast precursor - Arabidopsis thaliana (Mouse-ear cress), partial (25%) |
|  | GGAATGAGCGTGTTGGAAAAG | TC10229 | 3 | 5.815 | similar to UniRef100_P42738 Cluster: Chorismate mutase, chloroplast precursor; n=2; Arabidopsis thaliana\|Rep: Chorismate mutase, chloroplast precursor - Arabidopsis thaliana (Mouse-ear cress), partial (25%) |
|  | GGAATGAGCGTGTTGGAAAAGA | DW345618 | 3 | 5.058 |  |
|  | GGAATGAGCGTGTTGGAAAAGAA | DW345618 | 3 | 5.058 |  |
|  | TATTTTCTAAGCCTACTGTC | TC15592 | 2.5 | 20.472 | similar to UniRef100_A7QF28 Cluster: Chromosome chr16 scaffold_86, whole genome shotgun sequence; n=1; Vitis vinifera\|Rep: Chromosome chr16 scaffold_86, whole genome shotgun sequence - Vitis vinifera (Grape), partial (93%) |
|  | TCTAAGCCTACTGTCTTTCCC | DW346176 | 3 | 22.421 |  |
|  | TCTAAGCCTACTGTCTTTCCCT | DW346176 | 3 | 22.421 |  |
|  | TGCCAACCCCGCCCATTCCA | DW343104 | 3 | 24.615 | weakly similar to UniRef100_UPI0000E1F507 Cluster: PREDICTED: atonal homolog 8; n=1; Pan troglodytes\|Rep: PREDICTED: atonal homolog 8 - Pan troglodytes, partial (8%) |
|  | TGCCAACCCCGCCCATTCCAA | DW343104 | 3 | 24.615 | weakly similar to UniRef100_UPI0000E1F507 Cluster: PREDICTED: atonal homolog 8; n=1; Pan troglodytes\|Rep: PREDICTED: atonal homolog 8 - Pan troglodytes, partial (8%) |
|  | TGCCAACCCCGCCCATTCCAAA | DW343104 | 3 | 24.615 | weakly similar to UniRef100_UPI0000E1F507 Cluster: PREDICTED: atonal homolog 8; n=1; Pan troglodytes\|Rep: PREDICTED: atonal homolog 8 - Pan troglodytes, partial (8%) |
|  | TGGAATGAGCGTGTTGGAAAA | DW345618 | 2 | 5.064 |  |
|  | TTCTTTGCCAACCCCGCCCATT | DW341802 | 3 | 15.335 |  |
|  | TTGCCAACCCCGCCCATT |  |  |  |  |
|  | TTGCCAACCCCGCCCATTC |  |  |  |  |
|  | TTGCCAACCCCGCCCATTCC |  |  |  |  |
|  | TTGCCAACCCCGCCCATTCCA |  |  |  |  |
|  | TTGCCAACCCCGCCCATTCCAA |  |  |  |  |
|  | TTGCCAACCCCGCCCATTCCAAA |  |  |  |  |
|  | TTGCCAACCCCGCCCATTCCAAAT |  |  |  |  |
|  | TTTGAAGCAGATGATGGAAC | DW341292 | 2 | 13.845 | weakly similar to UniRef100_A1RKB5 Cluster: LPXTG-motif cell wall anchor domain precursor; n=1; Shewanella sp. W3-18-1\|Rep: LPXTG-motif cell wall anchor domain precursor - Shewanella sp. (strain W3-18-1), partial (3%) |
|  | TTTGCCAACCCCGCCCAT |  |  |  |  |
|  | TTTGCCAACCCCGCCCATT |  |  |  |  |
|  | TTTGCCAACCCCGCCCATTC |  |  |  |  |
|  | TTTGCCAACCCCGCCCATTCC |  |  |  |  |
|  | TTTGCCAACCCCGCCCATTCCA |  |  |  |  |
|  | TTTGCCAACCCCGCCCATTCCAA |  |  |  |  |
|  | TTTGCCAACCCCGCCCATTCCAAA |  |  |  |  |
|  |  |  |  |  |  |
| **1_29** | AGGTGGGCATACTGCCAACTG | AJ533494 | 2.5 | 19.316 |  |
|  | ATTGGCATTCTGTCCACCTCC | TC9507 | 2 | 16.562 | homologue to UniRef100_A7QP47 Cluster: Chromosome chr1 scaffold_136, whole genome shotgun sequence; n=1; Vitis vinifera\|Rep: Chromosome chr1 scaffold_136, whole genome shotgun sequence - Vitis vinifera (Grape), partial (40%) |
|  | TGGCATTCTGTCCACCTCC |  |  |  |  |
|  | TTGGCATTCTGTCCACCT | TC9507 | 1 | 15.772 | homologue to UniRef100_A7QP47 Cluster: Chromosome chr1 scaffold_136, whole genome shotgun sequence; n=1; Vitis vinifera\|Rep: Chromosome chr1 scaffold_136, whole genome shotgun sequence - Vitis vinifera (Grape), partial (40%) |
|  | TTGGCATTCTGTCCACCTC | TC9507 | 1 | 15.772 | homologue to UniRef100_A7QP47 Cluster: Chromosome chr1 scaffold_136, whole genome shotgun sequence; n=1; Vitis vinifera\|Rep: Chromosome chr1 scaffold_136, whole genome shotgun sequence - Vitis vinifera (Grape), partial (40%) |
|  | TTGGCATTCTGTCCACCTCC | TC9507 | 1 | 15.772 | homologue to UniRef100_A7QP47 Cluster: Chromosome chr1 scaffold_136, whole genome shotgun sequence; n=1; Vitis vinifera\|Rep: Chromosome chr1 scaffold_136, whole genome shotgun sequence - Vitis vinifera (Grape), partial (40%) |
|  | TTGGCATTCTGTCCACCTCCT | TC9507 | 1 | 15.772 | homologue to UniRef100_A7QP47 Cluster: Chromosome chr1 scaffold_136, whole genome shotgun sequence; n=1; Vitis vinifera\|Rep: Chromosome chr1 scaffold_136, whole genome shotgun sequence - Vitis vinifera (Grape), partial (40%) |
|  | TTGGCATTCTGTCCACCTCCTC | TC9507 | 1 | 15.772 | homologue to UniRef100_A7QP47 Cluster: Chromosome chr1 scaffold_136, whole genome shotgun sequence; n=1; Vitis vinifera\|Rep: Chromosome chr1 scaffold_136, whole genome shotgun sequence - Vitis vinifera (Grape), partial (40%) |
|  |  |  |  |  |  |
| **1_3** | AACATGATCATCCGAATGAT | TC16815 | 3 | 11.489 | similar to UniRef100_A8MRW0 Cluster: Uncharacterized protein At1g16240.3; n=1; Arabidopsis thaliana\|Rep: Uncharacterized protein At1g16240.3 - Arabidopsis thaliana (Mouse-ear cress), partial (76%) |
|  | AATGCTGTCTGGTTCGAGA |  |  |  |  |
|  | ACCAGGCTTCATTCCCCC |  |  |  |  |
|  | ATCCGAATGATCTCGGACCAGGCT | DY636551 | 3 | 15.597 | similar to UniRef100_A7P8C1 Cluster: Chromosome chr3 scaffold_8, whole genome shotgun sequence; n=1; Vitis vinifera\|Rep: Chromosome chr3 scaffold_8, whole genome shotgun sequence - Vitis vinifera (Grape), partial (80%) |
|  | ATCTCGGACCAGGCTTCATTCCCC |  |  |  |  |
|  | ATGCTGTCTGGTTCGAGA |  |  |  |  |
|  | CGGACCAGGCTTCATTCC |  |  |  |  |
|  | CGGACCAGGCTTCATTCCC |  |  |  |  |
|  | CGGACCAGGCTTCATTCCCC | FC862354 | 3 | 17.607 | weakly similar to UniRef100_Q93ZH4 Cluster: AT5g66850/MUD21_11; n=1; Arabidopsis thaliana\|Rep: AT5g66850/MUD21_11 - Arabidopsis thaliana (Mouse-ear cress), partial (9%) |
|  | CGGACCAGGCTTCATTCCCCC | FC862354 | 3 | 17.607 | weakly similar to UniRef100_Q93ZH4 Cluster: AT5g66850/MUD21_11; n=1; Arabidopsis thaliana\|Rep: AT5g66850/MUD21_11 - Arabidopsis thaliana (Mouse-ear cress), partial (9%) |
|  | CTCGGACCAGGCTTCATTCC |  |  |  |  |
|  | CTCGGACCAGGCTTCATTCCC |  |  |  |  |
|  | CTCGGACCAGGCTTCATTCCCC |  |  |  |  |
|  | CTCGGACCAGGCTTCATTCCCCC |  |  |  |  |
|  | GAATGCTGTCTGGTTCGAGAC |  |  |  |  |
|  | GACCAGGCTTCATTCCCC |  |  |  |  |
|  | GGAATGCTGTCTGGTTCGA |  |  |  |  |
|  | GGAATGCTGTCTGGTTCGAGA | TC12232 | 2.5 | 19.798 | similar to UniRef100_A7P229 Cluster: Chromosome chr19 scaffold_4, whole genome shotgun sequence; n=2; Vitis vinifera\|Rep: Chromosome chr19 scaffold_4, whole genome shotgun sequence - Vitis vinifera (Grape), partial (57%) |
|  | GGAATGCTGTCTGGTTCGAGAC |  |  |  |  |
|  | GGACCAGGCTTCATTCCC |  |  |  |  |
|  | GGACCAGGCTTCATTCCCC |  |  |  |  |
|  | TCGGACCAGGCTTCATTC |  |  |  |  |
|  | TCGGACCAGGCTTCATTCC |  |  |  |  |
|  | TCGGACCAGGCTTCATTCCC |  |  |  |  |
|  | TCGGACCAGGCTTCATTCCCC |  |  |  |  |
|  | TCGGACCAGGCTTCATTCCCCC |  |  |  |  |
|  | TCTCGGACCAGGCTTCATTCC | AJ826752 | 3 | 20.494 | similar to UniRef100_A7P6L8 Cluster: Chromosome chr9 scaffold_7, whole genome shotgun sequence; n=1; Vitis vinifera\|Rep: Chromosome chr9 scaffold_7, whole genome shotgun sequence - Vitis vinifera (Grape), partial (70%) |
|  | TCTCGGACCAGGCTTCATTCCC | AJ826752 | 3 | 20.494 | similar to UniRef100_A7P6L8 Cluster: Chromosome chr9 scaffold_7, whole genome shotgun sequence; n=1; Vitis vinifera\|Rep: Chromosome chr9 scaffold_7, whole genome shotgun sequence - Vitis vinifera (Grape), partial (70%) |
|  | TCTCGGACCAGGCTTCATTCCCC | AJ826752 | 3 | 20.494 | similar to UniRef100_A7P6L8 Cluster: Chromosome chr9 scaffold_7, whole genome shotgun sequence; n=1; Vitis vinifera\|Rep: Chromosome chr9 scaffold_7, whole genome shotgun sequence - Vitis vinifera (Grape), partial (70%) |
|  | TCTCGGACCAGGCTTCATTCCCCC | AJ826752 | 3 | 20.494 | similar to UniRef100_A7P6L8 Cluster: Chromosome chr9 scaffold_7, whole genome shotgun sequence; n=1; Vitis vinifera\|Rep: Chromosome chr9 scaffold_7, whole genome shotgun sequence - Vitis vinifera (Grape), partial (70%) |
|  |  |  |  |  |  |
| **1_32** | ATTGACAGAAGAGAGTGAGCAC | TC14700 | 3 | 15.068 | similar to UniRef100_A7PXQ8 Cluster: Chromosome chr15 scaffold_37, whole genome shotgun sequence; n=1; Vitis vinifera\|Rep: Chromosome chr15 scaffold_37, whole genome shotgun sequence - Vitis vinifera (Grape), partial (34%) |
|  | GACAGAAGAGAGTGAGCAC |  |  |  |  |
|  | GCTCATGTCTCTTTCTGTCAGC | DY639699 | 3 | 24.434 | similar to UniRef100_A6ZPM6 Cluster: Sit4 associated protein; n=1; Saccharomyces cerevisiae YJM789\|Rep: Sit4 associated protein, partial (1%) |
|  | GCTCATGTCTCTTTCTGTCAGCT | DY639699 | 3 | 24.434 | similar to UniRef100_A6ZPM6 Cluster: Sit4 associated protein; n=1; Saccharomyces cerevisiae YJM789\|Rep: Sit4 associated protein, partial (1%) |
|  | TGACAGAAGAGAGTGAGCA |  |  |  |  |
|  | TGACAGAAGAGAGTGAGCAC | DY638783 | 2.5 | 18.898 | similar to UniRef100_A7NXB7 Cluster: Chromosome chr5 scaffold_2, whole genome shotgun sequence; n=1; Vitis vinifera\|Rep: Chromosome chr5 scaffold_2, whole genome shotgun sequence - Vitis vinifera (Grape), partial (79%) |
|  | TGACAGAAGAGAGTGAGCACA | TC12590 | 2.5 | 19.819 | similar to UniRef100_A7Q1U2 Cluster: Chromosome chr7 scaffold_44, whole genome shotgun sequence; n=1; Vitis vinifera\|Rep: Chromosome chr7 scaffold_44, whole genome shotgun sequence - Vitis vinifera (Grape), partial (98%) |
|  | TGCTCATGTCTCTTTCTGTCAGC | DW346612 | 2.5 | 11.087 | weakly similar to UniRef100_A9BL23 Cluster: Nmt1; n=1; Rhodomonas salina\|Rep: Nmt1 - Rhodomonas salina (Cryptomonas salina), partial (8%) |
|  | TTGACAGAAGAGAGTGAGCAC | TC14700 | 3 | 15.129 | similar to UniRef100_A7PXQ8 Cluster: Chromosome chr15 scaffold_37, whole genome shotgun sequence; n=1; Vitis vinifera\|Rep: Chromosome chr15 scaffold_37, whole genome shotgun sequence - Vitis vinifera (Grape), partial (34%) |
|  |  |  |  |  |  |
| **1_44** | AGGTGGTCAGCATGTCAAACT | BU047479 | 3 | 20.587 | similar to UniRef100_A7Q448 Cluster: Chromosome chr13 scaffold_48, whole genome shotgun sequence; n=1; Vitis vinifera\|Rep: Chromosome chr13 scaffold_48, whole genome shotgun sequence - Vitis vinifera (Grape), partial (24%) |
|  | TGGCATTCTGTCCACCTCC |  |  |  |  |
|  | TTGGCATTCTGTCCACCT |  |  |  |  |
|  | TTGGCATTCTGTCCACCTC |  |  |  |  |
|  | TTGGCATTCTGTCCACCTCC | TC9507 | 1 | 15.772 | homologue to UniRef100_A7QP47 Cluster: Chromosome chr1 scaffold_136, whole genome shotgun sequence; n=1; Vitis vinifera\|Rep: Chromosome chr1 scaffold_136, whole genome shotgun sequence - Vitis vinifera (Grape), partial (40%) |
|  | TTTGGCATTCTGTCCACCTCC | TC9507 | 1 | 16.562 | homologue to UniRef100_A7QP47 Cluster: Chromosome chr1 scaffold_136, whole genome shotgun sequence; n=1; Vitis vinifera\|Rep: Chromosome chr1 scaffold_136, whole genome shotgun sequence - Vitis vinifera (Grape), partial (40%) |
|  |  |  |  |  |  |
| **1_5** | TACAATGAAATCACGGCC |  |  |  |  |
|  | TATAAAGAGATGTACTGGACC | TC9878 | 3 | 19.827 | similar to UniRef100_Q6QLW8 Cluster: HMG-CoA synthase 2; n=1; Hevea brasiliensis\|Rep: HMG-CoA synthase 2 - Hevea brasiliensis (Para rubber tree), partial (35%) |
|  | TTATACAATGAAATCACGG |  |  |  |  |
|  | TTATACAATGAAATCACGGC | TC16834 | 3 | 14.203 | similar to UniRef100_Q9M385 Cluster: 50S ribosomal protein L17, chloroplast precursor; n=1; Arabidopsis thaliana\|Rep: 50S ribosomal protein L17, chloroplast precursor - Arabidopsis thaliana (Mouse-ear cress), partial (73%) |
|  | TTATACAATGAAATCACGGCC | TC16834 | 3 | 14.203 | similar to UniRef100_Q9M385 Cluster: 50S ribosomal protein L17, chloroplast precursor; n=1; Arabidopsis thaliana\|Rep: 50S ribosomal protein L17, chloroplast precursor - Arabidopsis thaliana (Mouse-ear cress), partial (73%) |
|  | TTATACAATGAAATCACGGCCG | TC16834 | 3 | 14.203 | similar to UniRef100_Q9M385 Cluster: 50S ribosomal protein L17, chloroplast precursor; n=1; Arabidopsis thaliana\|Rep: 50S ribosomal protein L17, chloroplast precursor - Arabidopsis thaliana (Mouse-ear cress), partial (73%) |
|  |  |  |  |  |  |
| **10_1** | ACAGGGAACAGGTAGAGCA |  |  |  |  |
|  | ACAGGGAACAGGTAGAGCATG | TC8727 | 3 | 15.603 | homologue to UniRef100_Q2Z1Y2 Cluster: Serine carboxypeptidase; n=1; Prunus mume\|Rep: Serine carboxypeptidase - Prunus mume (Japanese flowering apricot), partial (47%) |
|  | ATGCACTGCCTCTTCCCTGGC | TC12296 | 3 | 20.159 | homologue to UniRef100_A7PEX6 Cluster: Chromosome chr11 scaffold_13, whole genome shotgun sequence; n=1; Vitis vinifera\|Rep: Chromosome chr11 scaffold_13, whole genome shotgun sequence - Vitis vinifera (Grape), partial (92%) |
|  | TGCACTGCCTCTTCCCTG |  |  |  |  |
|  | TGCACTGCCTCTTCCCTGG |  |  |  |  |
|  | TGCACTGCCTCTTCCCTGGC | FC867363 | 3 | 23.667 | similar to UniRef100_A7PLG7 Cluster: Chromosome chr7 scaffold_20, whole genome shotgun sequence; n=1; Vitis vinifera\|Rep: Chromosome chr7 scaffold_20, whole genome shotgun sequence - Vitis vinifera (Grape), partial (31%) |
|  | TGCACTGCCTCTTCCCTGGCT | FC867363 | 3 | 23.667 | similar to UniRef100_A7PLG7 Cluster: Chromosome chr7 scaffold_20, whole genome shotgun sequence; n=1; Vitis vinifera\|Rep: Chromosome chr7 scaffold_20, whole genome shotgun sequence - Vitis vinifera (Grape), partial (31%) |
|  | TGCACTGCCTCTTCCCTGGCTG | FC867363 | 3 | 23.667 | similar to UniRef100_A7PLG7 Cluster: Chromosome chr7 scaffold_20, whole genome shotgun sequence; n=1; Vitis vinifera\|Rep: Chromosome chr7 scaffold_20, whole genome shotgun sequence - Vitis vinifera (Grape), partial (31%) |
|  |  |  |  |  |  |
| **2_31** | CCAAAGGGATCGCATTGATCT | TC11240 | 3 | 22.032 | similar to UniRef100_A4KA30 Cluster: Transport inhibitor response 1; n=1; Gossypium hirsutum\|Rep: Transport inhibitor response 1 - Gossypium hirsutum (Upland cotton) (Gossypium mexicanum), partial (37%) |
|  | TCCAAAGGGATCGCATTGA |  |  |  |  |
|  | TCCAAAGGGATCGCATTGAT | TC11240 | 2 | 19.247 | similar to UniRef100_A4KA30 Cluster: Transport inhibitor response 1; n=1; Gossypium hirsutum\|Rep: Transport inhibitor response 1 - Gossypium hirsutum (Upland cotton) (Gossypium mexicanum), partial (37%) |
|  | TCCAAAGGGATCGCATTGATC | TC11240 | 2 | 19.247 | similar to UniRef100_A4KA30 Cluster: Transport inhibitor response 1; n=1; Gossypium hirsutum\|Rep: Transport inhibitor response 1 - Gossypium hirsutum (Upland cotton) (Gossypium mexicanum), partial (37%) |
|  | TCCAAAGGGATCGCATTGATCT | TC11240 | 2 | 19.247 | similar to UniRef100_A4KA30 Cluster: Transport inhibitor response 1; n=1; Gossypium hirsutum\|Rep: Transport inhibitor response 1 - Gossypium hirsutum (Upland cotton) (Gossypium mexicanum), partial (37%) |
|  | TCCAAAGGGATCGCATTGATCTA | TC11240 | 2 | 19.247 | similar to UniRef100_A4KA30 Cluster: Transport inhibitor response 1; n=1; Gossypium hirsutum\|Rep: Transport inhibitor response 1 - Gossypium hirsutum (Upland cotton) (Gossypium mexicanum), partial (37%) |
|  | TCGATGCGATCCCTTGGGA |  |  |  |  |
|  | TCGATGCGATCCCTTGGGAAG | TC9374 | 3 | 23.312 | weakly similar to UniRef100_Q41321 Cluster: Protein induced upon tuberization; n=1; Solanum demissum\|Rep: Protein induced upon tuberization - Solanum demissum (Wild potato), partial (78%) |
|  | TCGATGCGATCCCTTGGGAAGT | TC9374 | 3 | 23.312 | weakly similar to UniRef100_Q41321 Cluster: Protein induced upon tuberization; n=1; Solanum demissum\|Rep: Protein induced upon tuberization - Solanum demissum (Wild potato), partial (78%) |
|  | TGATATTGGATCGATGCGATC | DY653623 | 3 | 20.33 | homologue to UniRef100_Q6UNT3 Cluster: Hypersensitive-induced response protein; n=1; Cucumis sativus\|Rep: Hypersensitive-induced response protein - Cucumis sativus (Cucumber), partial (48%) |
|  |  |  |  |  |  |
| **3_16** | ATTGTAGGAATGGGCTGTTTG |  |  |  |  |
|  | CCCAAGCCCGCCCATTCC |  |  |  |  |
|  | CCCAAGCCCGCCCATTCCA |  |  |  |  |
|  | CTTCCCAAGCCCGCCCATTCCA | DW341459 | 2 | 17.556 | similar to UniRef100_Q9LX66 Cluster: Probable receptor-like protein kinase At3g46290 precursor; n=1; Arabidopsis thaliana\|Rep: Probable receptor-like protein kinase At3g46290 precursor - Arabidopsis thaliana (Mouse-ear cress), partial (26%) |
|  | GGAATGGGCTGTTTGGGA |  |  |  |  |
|  | GGAATGGGCTGTTTGGGAT |  |  |  |  |
|  | GGAATGGGCTGTTTGGGATG | TC15058 | 3 | 11.735 | homologue to UniRef100_Q7QEA4 Cluster: AGAP000748-PA; n=1; Anopheles gambiae str. PEST\|Rep: AGAP000748-PA - Anopheles gambiae str. PEST, partial (13%) |
|  | GGAATGGGCTGTTTGGGATGA | TC15058 | 3 | 11.735 | homologue to UniRef100_Q7QEA4 Cluster: AGAP000748-PA; n=1; Anopheles gambiae str. PEST\|Rep: AGAP000748-PA - Anopheles gambiae str. PEST, partial (13%) |
|  | GGAATGGGCTGTTTGGGATGAA | TC15058 | 3 | 11.735 | homologue to UniRef100_Q7QEA4 Cluster: AGAP000748-PA; n=1; Anopheles gambiae str. PEST\|Rep: AGAP000748-PA - Anopheles gambiae str. PEST, partial (13%) |
|  | GGAATGGGCTGTTTGGGATGAAAG | TC12108 | 3 | 20.431 | homologue to UniRef100_A7P081 Cluster: Chromosome chr6 scaffold_3, whole genome shotgun sequence; n=1; Vitis vinifera\|Rep: Chromosome chr6 scaffold_3, whole genome shotgun sequence - Vitis vinifera (Grape), complete |
|  | TAGGAATGGGCTGTTTGGGA |  |  |  |  |
|  | TTCCCAAGCCCGCCCATT |  |  |  |  |
|  | TTCCCAAGCCCGCCCATTC |  |  |  |  |
|  | TTCCCAAGCCCGCCCATTCC | DW341459 | 2 | 17.288 | similar to UniRef100_Q9LX66 Cluster: Probable receptor-like protein kinase At3g46290 precursor; n=1; Arabidopsis thaliana\|Rep: Probable receptor-like protein kinase At3g46290 precursor - Arabidopsis thaliana (Mouse-ear cress), partial (26%) |
|  | TTCCCAAGCCCGCCCATTCCA | DW341459 | 2 | 17.288 | similar to UniRef100_Q9LX66 Cluster: Probable receptor-like protein kinase At3g46290 precursor; n=1; Arabidopsis thaliana\|Rep: Probable receptor-like protein kinase At3g46290 precursor - Arabidopsis thaliana (Mouse-ear cress), partial (26%) |
|  | TTCCCAAGCCCGCCCATTCCAA | DW341459 | 2 | 17.288 | similar to UniRef100_Q9LX66 Cluster: Probable receptor-like protein kinase At3g46290 precursor; n=1; Arabidopsis thaliana\|Rep: Probable receptor-like protein kinase At3g46290 precursor - Arabidopsis thaliana (Mouse-ear cress), partial (26%) |
|  | TTGTAGGAATGGGCTGTTTGGGA | TC10070 | 3 | 19.582 | similar to UniRef100_Q000L0 Cluster: Acetyl-CoA carboxylase; n=1; Jatropha curcas\|Rep: Acetyl-CoA carboxylase - Jatropha curcas, partial (19%) |
|  | TTTCTTTCATCCCAAACAGCC | TC16634 | 2.5 | 20.628 | similar to UniRef100_A7QS50 Cluster: Chromosome chr5 scaffold_156, whole genome shotgun sequence; n=1; Vitis vinifera\|Rep: Chromosome chr5 scaffold_156, whole genome shotgun sequence - Vitis vinifera (Grape), partial (9%) |
|  |  |  |  |  |  |
| **3_28** | ATGGTGTCATCCCTCCTGTGACC | TC11677 | 3 | 23.5 | weakly similar to UniRef100_A7QWL4 Cluster: Chromosome chr4 scaffold_205, whole genome shotgun sequence; n=1; Vitis vinifera\|Rep: Chromosome chr4 scaffold_205, whole genome shotgun sequence - Vitis vinifera (Grape), partial (79%) |
|  | CCAAATTGAGAGAGAGAGAGAGAG | TC13590 | 1.5 | 5.962 | similar to UniRef100_A7PPC9 Cluster: Chromosome chr8 scaffold_23, whole genome shotgun sequence; n=1; Vitis vinifera\|Rep: Chromosome chr8 scaffold_23, whole genome shotgun sequence - Vitis vinifera (Grape), partial (56%) |
|  | CCATCTTCCTGTGACATGAAC | DW345200 | 3 | 22.741 |  |
|  | CGCAGGAGAGATGGCACTG |  |  |  |  |
|  | GGTGTCATCCCTCCTGTGACC |  |  |  |  |
|  | TCCATCTTCCTGTGACATGA | TC9564 | 3 | 10.28 | similar to UniRef100_A7QR34 Cluster: Chromosome undetermined scaffold_147, whole genome shotgun sequence; n=1; Vitis vinifera\|Rep: Chromosome undetermined scaffold_147, whole genome shotgun sequence - Vitis vinifera (Grape), partial (23%) |
|  | TCGCAGGAGAGATGGCAC |  |  |  |  |
|  | TCGCAGGAGAGATGGCACTG |  |  |  |  |
|  | TCGCAGGAGAGATGGCACTGT |  |  |  |  |
|  | TCGCAGGAGAGATGGCACTGTC |  |  |  |  |
|  | TCGCAGGAGAGATGGCACTGTCT |  |  |  |  |
|  | TGGTGTCATCCCTCCTGTGACC |  |  |  |  |
|  | TTCCATCTTCCTGTGACATGA | TC15200 | 3 | 13.366 | similar to UniRef100_A7Q3T3 Cluster: Chromosome chr13 scaffold_48, whole genome shotgun sequence; n=1; Vitis vinifera\|Rep: Chromosome chr13 scaffold_48, whole genome shotgun sequence - Vitis vinifera (Grape), partial (89%) |
|  | TTCGCAGGAGAGATGGCAC |  |  |  |  |
|  | TTCGCAGGAGAGATGGCACTGTC | TC11826 | 3 | 20.79 | similar to UniRef100_A7PZF0 Cluster: Chromosome chr15 scaffold_40, whole genome shotgun sequence; n=2; Vitis vinifera\|Rep: Chromosome chr15 scaffold_40, whole genome shotgun sequence - Vitis vinifera (Grape), partial (52%) |
|  |  |  |  |  |  |
| **4_21** | CCCTGCAGTACCTTCCTTTACCC |  |  |  |  |
|  | GGAGCGACCTGGGATCACATG |  |  |  |  |
|  | GTGTTCTCAGGTCGCCCCTG | BU039242 | 3 | 16.719 | homologue to UniRef100_A7PD59 Cluster: Chromosome chr17 scaffold_12, whole genome shotgun sequence; n=1; Vitis vinifera\|Rep: Chromosome chr17 scaffold_12, whole genome shotgun sequence - Vitis vinifera (Grape), partial (70%) |
|  | TGTGTTCTCAGGTCGCCCC |  |  |  |  |
|  | TGTGTTCTCAGGTCGCCCCT | FC862607 | 3 | 24.183 | weakly similar to UniRef100_A7PNX0 Cluster: Chromosome chr8 scaffold_23, whole genome shotgun sequence; n=1; Vitis vinifera\|Rep: Chromosome chr8 scaffold_23, whole genome shotgun sequence - Vitis vinifera (Grape), partial (32%) |
|  | TGTGTTCTCAGGTCGCCCCTG | FC862607 | 3 | 24.183 | weakly similar to UniRef100_A7PNX0 Cluster: Chromosome chr8 scaffold_23, whole genome shotgun sequence; n=1; Vitis vinifera\|Rep: Chromosome chr8 scaffold_23, whole genome shotgun sequence - Vitis vinifera (Grape), partial (32%) |
|  |  |  |  |  |  |
| **5_14** | AATGTTGTCTGGCTCGAG |  |  |  |  |
|  | AATGTTGTCTGGCTCGAGG |  |  |  |  |
|  | AATGTTGTCTGGCTCGAGGCC | BU043275 | 2.5 | 15.034 | weakly similar to UniRef100_Q94AD6 Cluster: AT5g24810/F6A4_20; n=1; Arabidopsis thaliana\|Rep: AT5g24810/F6A4_20 - Arabidopsis thaliana (Mouse-ear cress), partial (24%) |
|  | AATGTTGTCTGGCTCGAGGCCC | BU043275 | 2.5 | 15.034 | weakly similar to UniRef100_Q94AD6 Cluster: AT5g24810/F6A4_20; n=1; Arabidopsis thaliana\|Rep: AT5g24810/F6A4_20 - Arabidopsis thaliana (Mouse-ear cress), partial (24%) |
|  | AATGTTGTCTGGCTCGAGGCCCCT | BU043275 | 2.5 | 15.034 | weakly similar to UniRef100_Q94AD6 Cluster: AT5g24810/F6A4_20; n=1; Arabidopsis thaliana\|Rep: AT5g24810/F6A4_20 - Arabidopsis thaliana (Mouse-ear cress), partial (24%) |
|  | ACCAGGCTTCATTCCCCC |  |  |  |  |
|  | ACGTCGGACCAGGCTTCATTC |  |  |  |  |
|  | ACGTCGGACCAGGCTTCATTCCCC |  |  |  |  |
|  | ATGTTGTCTGGCTCGAGG |  |  |  |  |
|  | ATTTGGTTCTACATTTAGTGAC | DW343084 | 2.5 | 10.04 | UniRef100_A8M4Y5 Cluster: Major facilitator superfamily MFS_1; n=1; Salinispora arenicola CNS-205\|Rep: Major, partial (1%) |
|  | CGGACCAGGCTTCATTCC |  |  |  |  |
|  | CGGACCAGGCTTCATTCCC |  |  |  |  |
|  | CGGACCAGGCTTCATTCCCC | FC862354 | 3 | 17.607 | weakly similar to UniRef100_Q93ZH4 Cluster: AT5g66850/MUD21_11; n=1; Arabidopsis thaliana\|Rep: AT5g66850/MUD21_11 - Arabidopsis thaliana (Mouse-ear cress), partial (9%) |
|  | CGGACCAGGCTTCATTCCCCC | FC862354 | 3 | 17.607 | weakly similar to UniRef100_Q93ZH4 Cluster: AT5g66850/MUD21_11; n=1; Arabidopsis thaliana\|Rep: AT5g66850/MUD21_11 - Arabidopsis thaliana (Mouse-ear cress), partial (9%) |
|  | CGTCGGACCAGGCTTCATTCC |  |  |  |  |
|  | CGTCGGACCAGGCTTCATTCCC |  |  |  |  |
|  | CGTCGGACCAGGCTTCATTCCCC |  |  |  |  |
|  | GAATGTTGTCTGGCTCGA |  |  |  |  |
|  | GAATGTTGTCTGGCTCGAGG | BU043275 | 2.5 | 12.654 | weakly similar to UniRef100_Q94AD6 Cluster: AT5g24810/F6A4_20; n=1; Arabidopsis thaliana\|Rep: AT5g24810/F6A4_20 - Arabidopsis thaliana (Mouse-ear cress), partial (24%) |
|  | GAATGTTGTCTGGCTCGAGGC | BU043275 | 2.5 | 12.654 | weakly similar to UniRef100_Q94AD6 Cluster: AT5g24810/F6A4_20; n=1; Arabidopsis thaliana\|Rep: AT5g24810/F6A4_20 - Arabidopsis thaliana (Mouse-ear cress), partial (24%) |
|  | GAATGTTGTCTGGCTCGAGGCC | BU043275 | 2.5 | 12.654 | weakly similar to UniRef100_Q94AD6 Cluster: AT5g24810/F6A4_20; n=1; Arabidopsis thaliana\|Rep: AT5g24810/F6A4_20 - Arabidopsis thaliana (Mouse-ear cress), partial (24%) |
|  | GAATGTTGTCTGGCTCGAGGCCCC | BU043275 | 2.5 | 12.654 | weakly similar to UniRef100_Q94AD6 Cluster: AT5g24810/F6A4_20; n=1; Arabidopsis thaliana\|Rep: AT5g24810/F6A4_20 - Arabidopsis thaliana (Mouse-ear cress), partial (24%) |
|  | GACCAGGCTTCATTCCCC |  |  |  |  |
|  | GGAATGTTGTCTGGCTCG |  |  |  |  |
|  | GGAATGTTGTCTGGCTCGA |  |  |  |  |
|  | GGAATGTTGTCTGGCTCGAG | TC15757 | 3 | 20.094 | similar to UniRef100_A7QHZ9 Cluster: Chromosome chr17 scaffold_101, whole genome shotgun sequence; n=1; Vitis vinifera\|Rep: Chromosome chr17 scaffold_101, whole genome shotgun sequence - Vitis vinifera (Grape), partial (81%) |
|  | GGAATGTTGTCTGGCTCGAGG | TC15757 | 3 | 20.094 | similar to UniRef100_A7QHZ9 Cluster: Chromosome chr17 scaffold_101, whole genome shotgun sequence; n=1; Vitis vinifera\|Rep: Chromosome chr17 scaffold_101, whole genome shotgun sequence - Vitis vinifera (Grape), partial (81%) |
|  | GGAATGTTGTCTGGCTCGAGGC | TC15757 | 3 | 20.094 | similar to UniRef100_A7QHZ9 Cluster: Chromosome chr17 scaffold_101, whole genome shotgun sequence; n=1; Vitis vinifera\|Rep: Chromosome chr17 scaffold_101, whole genome shotgun sequence - Vitis vinifera (Grape), partial (81%) |
|  | GGACCAGGCTTCATTCCC |  |  |  |  |
|  | GGACCAGGCTTCATTCCCC |  |  |  |  |
|  | GTCGGACCAGGCTTCATTC |  |  |  |  |
|  | GTCGGACCAGGCTTCATTCC | DY640430 | 3 | 19.419 | similar to UniRef100_A7P3C4 Cluster: Chromosome chr1 scaffold_5, whole genome shotgun sequence; n=1; Vitis vinifera\|Rep: Chromosome chr1 scaffold_5, whole genome shotgun sequence - Vitis vinifera (Grape), partial (49%) |
|  | GTCGGACCAGGCTTCATTCCC | DY640430 | 3 | 19.419 | similar to UniRef100_A7P3C4 Cluster: Chromosome chr1 scaffold_5, whole genome shotgun sequence; n=1; Vitis vinifera\|Rep: Chromosome chr1 scaffold_5, whole genome shotgun sequence - Vitis vinifera (Grape), partial (49%) |
|  | GTCGGACCAGGCTTCATTCCCC | DY640430 | 3 | 19.419 | similar to UniRef100_A7P3C4 Cluster: Chromosome chr1 scaffold_5, whole genome shotgun sequence; n=1; Vitis vinifera\|Rep: Chromosome chr1 scaffold_5, whole genome shotgun sequence - Vitis vinifera (Grape), partial (49%) |
|  | GTCGGACCAGGCTTCATTCCCCC | DY640430 | 3 | 19.419 | similar to UniRef100_A7P3C4 Cluster: Chromosome chr1 scaffold_5, whole genome shotgun sequence; n=1; Vitis vinifera\|Rep: Chromosome chr1 scaffold_5, whole genome shotgun sequence - Vitis vinifera (Grape), partial (49%) |
|  | GTTGTCTGGCTCGAGGCC |  |  |  |  |
|  | TAAATGTAGAACCAAATGATCT | TC9340 | 3 | 20.499 | UniRef100_Q946X6 Cluster: Vacuolar H+-pyrophosphatase; n=1; Prunus persica\|Rep: Vacuolar H+-pyrophosphatase - Prunus persica (Peach), complete |
|  | TCACTAAATGTAGAACCAAATG | DW343938 | 2.5 | 13.763 |  |
|  | TCGGACCAGGCTTCATTC |  |  |  |  |
|  | TCGGACCAGGCTTCATTCC |  |  |  |  |
|  | TCGGACCAGGCTTCATTCCC |  |  |  |  |
|  | TCGGACCAGGCTTCATTCCCC |  |  |  |  |
|  | TCGGACCAGGCTTCATTCCCCC |  |  |  |  |
|  | TGTCTGGCTCGAGGCCCCTA |  |  |  |  |
|  |  |  |  |  |  |
| **5_3** | CCCGCCTTGCATCAACTG |  |  |  |  |
|  | CCCGCCTTGCATCAACTGAA |  |  |  |  |
|  | CCCGCCTTGCATCAACTGAAT |  |  |  |  |
|  | CCGCCTTGCATCAACTGAAT |  |  |  |  |
|  | CGCTTGGTGCAGGTCGGGA |  |  |  |  |
|  | CGCTTGGTGCAGGTCGGGAA | DY653024 | 3 | 17.768 | weakly similar to UniRef100_A7NYE0 Cluster: Chromosome chr6 scaffold_3, whole genome shotgun sequence; n=1; Vitis vinifera\|Rep: Chromosome chr6 scaffold_3, whole genome shotgun sequence - Vitis vinifera (Grape), partial (22%) |
|  | CGCTTGGTGCAGGTCGGGAAC | DY653024 | 3 | 17.768 | weakly similar to UniRef100_A7NYE0 Cluster: Chromosome chr6 scaffold_3, whole genome shotgun sequence; n=1; Vitis vinifera\|Rep: Chromosome chr6 scaffold_3, whole genome shotgun sequence - Vitis vinifera (Grape), partial (22%) |
|  | GCTTGGTGCAGGTCGGGAA |  |  |  |  |
|  | GGGTCCCGCCTTGCATCAAC |  |  |  |  |
|  | GGTCCCGCCTTGCATCAACTGAAT |  |  |  |  |
|  | TCGCTTGGTGCAGGTCGGGA | TC12995 | 3 | 22.946 | similar to UniRef100_A7PIX0 Cluster: Chromosome chr13 scaffold_17, whole genome shotgun sequence; n=1; Vitis vinifera\|Rep: Chromosome chr13 scaffold_17, whole genome shotgun sequence - Vitis vinifera (Grape), partial (67%) |
|  | TCGCTTGGTGCAGGTCGGGAA | TC12995 | 3 | 22.946 | similar to UniRef100_A7PIX0 Cluster: Chromosome chr13 scaffold_17, whole genome shotgun sequence; n=1; Vitis vinifera\|Rep: Chromosome chr13 scaffold_17, whole genome shotgun sequence - Vitis vinifera (Grape), partial (67%) |
|  | TCGCTTGGTGCAGGTCGGGAACT | TC12995 | 3 | 22.946 | similar to UniRef100_A7PIX0 Cluster: Chromosome chr13 scaffold_17, whole genome shotgun sequence; n=1; Vitis vinifera\|Rep: Chromosome chr13 scaffold_17, whole genome shotgun sequence - Vitis vinifera (Grape), partial (67%) |
|  | TGGGTCCCGCCTTGCATCAAC |  |  |  |  |
|  | TGGGTCCCGCCTTGCATCAACT |  |  |  |  |
|  | TGGTGCAGGTCGGGAACTGCT | TC14169 | 3 | 21.393 | similar to UniRef100_A7Q1C4 Cluster: Chromosome chr10 scaffold_43, whole genome shotgun sequence; n=1; Vitis vinifera\|Rep: Chromosome chr10 scaffold_43, whole genome shotgun sequence - Vitis vinifera (Grape), partial (35%) |
|  | TTGGTCGGTGGGTGCGAAATGGGT |  |  |  |  |
|  |  |  |  |  |  |
| **6_29** | AAGCTCAGGAGGGATAGC |  |  |  |  |
|  | AAGCTCAGGAGGGATAGCGC | FC865206 | 2.5 | 19.818 | weakly similar to UniRef100_A7QDE5 Cluster: Chromosome chr10 scaffold_81, whole genome shotgun sequence; n=1; Vitis vinifera\|Rep: Chromosome chr10 scaffold_81, whole genome shotgun sequence - Vitis vinifera (Grape), partial (18%) |
|  | AAGCTCAGGAGGGATAGCGCC | FC865206 | 2.5 | 19.818 | weakly similar to UniRef100_A7QDE5 Cluster: Chromosome chr10 scaffold_81, whole genome shotgun sequence; n=1; Vitis vinifera\|Rep: Chromosome chr10 scaffold_81, whole genome shotgun sequence - Vitis vinifera (Grape), partial (18%) |
|  | AGCTCAGGAGGGATAGCGCC | TC10332 | 2.5 | 10.787 | similar to UniRef100_A7P396 Cluster: Chromosome chr1 scaffold_5, whole genome shotgun sequence; n=1; Vitis vinifera\|Rep: Chromosome chr1 scaffold_5, whole genome shotgun sequence - Vitis vinifera (Grape), partial (76%) |
|  | CGCTATCCATCCTGAGTTTC |  |  |  |  |
|  | CGCTATCCATCCTGAGTTTCA |  |  |  |  |
|  | TATTGCGCTATCCATCCTGAGTT |  |  |  |  |
|  | TCCATCCTGAGTTTCATGGCT | TC9666 | 2.5 | 16.342 | similar to UniRef100_A7PL53 Cluster: Chromosome chr7 scaffold_20, whole genome shotgun sequence; n=1; Vitis vinifera\|Rep: Chromosome chr7 scaffold_20, whole genome shotgun sequence - Vitis vinifera (Grape), partial (77%) |
|  | TTGCGCTATCCATCCTGAG |  |  |  |  |
|  |  |  |  |  |  |
| **6_30** | AAGCTGCCAGCATGATCTGAGC | TC16394 | 1.5 | 20.549 | similar to UniRef100_A7PTN9 Cluster: Chromosome undetermined scaffold_30, whole genome shotgun sequence; n=1; Vitis vinifera\|Rep: Chromosome undetermined scaffold_30, whole genome shotgun sequence - Vitis vinifera (Grape), partial (67%) |
|  | AGATCATGTGGTAGCTTCATC | TC10466 | 3 | 17.754 | similar to UniRef100_A7NYM9 Cluster: Chromosome chr6 scaffold_3, whole genome shotgun sequence; n=1; Vitis vinifera\|Rep: Chromosome chr6 scaffold_3, whole genome shotgun sequence - Vitis vinifera (Grape), partial (52%) |
|  | CTAGATCATGTGGTAGCTTCATC | FC862790 | 2 | 16.838 | similar to UniRef100_A7NVZ4 Cluster: Chromosome chr5 scaffold_2, whole genome shotgun sequence; n=1; Vitis vinifera\|Rep: Chromosome chr5 scaffold_2, whole genome shotgun sequence - Vitis vinifera (Grape), partial (52%) |
|  | GAAGCTGCCAGCATGATCTG | TC16394 | 2.5 | 22.259 | similar to UniRef100_A7PTN9 Cluster: Chromosome undetermined scaffold_30, whole genome shotgun sequence; n=1; Vitis vinifera\|Rep: Chromosome undetermined scaffold_30, whole genome shotgun sequence - Vitis vinifera (Grape), partial (67%) |
|  | GAAGCTGCCAGCATGATCTGA | TC16394 | 2.5 | 22.259 | similar to UniRef100_A7PTN9 Cluster: Chromosome undetermined scaffold_30, whole genome shotgun sequence; n=1; Vitis vinifera\|Rep: Chromosome undetermined scaffold_30, whole genome shotgun sequence - Vitis vinifera (Grape), partial (67%) |
|  | GATCATGTGGTAGCTTCATC | TC10466 | 3 | 19.525 | similar to UniRef100_A7NYM9 Cluster: Chromosome chr6 scaffold_3, whole genome shotgun sequence; n=1; Vitis vinifera\|Rep: Chromosome chr6 scaffold_3, whole genome shotgun sequence - Vitis vinifera (Grape), partial (52%) |
|  | GCTAGATCATGTGGTAGCTTCATC | FC862790 | 2 | 16.4 | similar to UniRef100_A7NVZ4 Cluster: Chromosome chr5 scaffold_2, whole genome shotgun sequence; n=1; Vitis vinifera\|Rep: Chromosome chr5 scaffold_2, whole genome shotgun sequence - Vitis vinifera (Grape), partial (52%) |
|  | TAGATCATGTGGTAGCTTCATC | FC862790 | 3 | 12.314 | similar to UniRef100_A7NVZ4 Cluster: Chromosome chr5 scaffold_2, whole genome shotgun sequence; n=1; Vitis vinifera\|Rep: Chromosome chr5 scaffold_2, whole genome shotgun sequence - Vitis vinifera (Grape), partial (52%) |
|  | TGAAGCTGCCAGCATGAT |  |  |  |  |
|  | TGAAGCTGCCAGCATGATC |  |  |  |  |
|  | TGAAGCTGCCAGCATGATCT | TC16861 | 3 | 17.365 | weakly similar to UniRef100_Q5YDB6 Cluster: C-terminal domain phosphatase-like 1; n=1; Arabidopsis thaliana\|Rep: C-terminal domain phosphatase-like 1 - Arabidopsis thaliana (Mouse-ear cress), partial (3%) |
|  | TGAAGCTGCCAGCATGATCTG | TC16861 | 3 | 17.365 | weakly similar to UniRef100_Q5YDB6 Cluster: C-terminal domain phosphatase-like 1; n=1; Arabidopsis thaliana\|Rep: C-terminal domain phosphatase-like 1 - Arabidopsis thaliana (Mouse-ear cress), partial (3%) |
|  | TGAAGCTGCCAGCATGATCTGA | TC16861 | 3 | 17.365 | weakly similar to UniRef100_Q5YDB6 Cluster: C-terminal domain phosphatase-like 1; n=1; Arabidopsis thaliana\|Rep: C-terminal domain phosphatase-like 1 - Arabidopsis thaliana (Mouse-ear cress), partial (3%) |
|  | TGAAGCTGCCAGCATGATCTGAGC | TC16861 | 3 | 17.365 | weakly similar to UniRef100_Q5YDB6 Cluster: C-terminal domain phosphatase-like 1; n=1; Arabidopsis thaliana\|Rep: C-terminal domain phosphatase-like 1 - Arabidopsis thaliana (Mouse-ear cress), partial (3%) |
|  | TGTTGAAGCTGCCAGCATGATC |  |  |  |  |
|  |  |  |  |  |  |
| **6_4** | AAGCTCAGGAGGGATAGC |  |  |  |  |
|  | AAGCTCAGGAGGGATAGCGC | FC865206 | 2.5 | 19.818 | weakly similar to UniRef100_A7QDE5 Cluster: Chromosome chr10 scaffold_81, whole genome shotgun sequence; n=1; Vitis vinifera\|Rep: Chromosome chr10 scaffold_81, whole genome shotgun sequence - Vitis vinifera (Grape), partial (18%) |
|  | AAGCTCAGGAGGGATAGCGCC | FC865206 | 2.5 | 19.818 | weakly similar to UniRef100_A7QDE5 Cluster: Chromosome chr10 scaffold_81, whole genome shotgun sequence; n=1; Vitis vinifera\|Rep: Chromosome chr10 scaffold_81, whole genome shotgun sequence - Vitis vinifera (Grape), partial (18%) |
|  | AGCTCAGGAGGGATAGCGCC | TC10332 | 2.5 | 10.787 | similar to UniRef100_A7P396 Cluster: Chromosome chr1 scaffold_5, whole genome shotgun sequence; n=1; Vitis vinifera\|Rep: Chromosome chr1 scaffold_5, whole genome shotgun sequence - Vitis vinifera (Grape), partial (76%) |
|  | CGCTATCTATCCTGAGTTTCA | TC15866 | 3 | 16.12 | similar to UniRef100_A7PJE3 Cluster: Chromosome chr12 scaffold_18, whole genome shotgun sequence; n=1; Vitis vinifera\|Rep: Chromosome chr12 scaffold_18, whole genome shotgun sequence - Vitis vinifera (Grape), partial (39%) |
|  |  |  |  |  |  |
| **6_7** | AATTACTACTTTTGAGTGGTTA |  |  |  |  |
|  | ATCTTTCCCAATCCACCCA |  |  |  |  |
|  | ATCTTTCCCAATCCACCCATGCC | AJ822927 | 3 | 24.773 |  |
|  | CATGGGTAAGTGGGGAAGA |  |  |  |  |
|  | CATGGGTAAGTGGGGAAGATG | DW346916 | 3 | 12.443 | similar to UniRef100_A9SNZ3 Cluster: Predicted protein; n=1; Physcomitrella patens subsp. patens\|Rep: Predicted protein - Physcomitrella patens subsp. patens, partial (3%) |
|  | CATGGGTAAGTGGGGAAGATGA | DW346916 | 3 | 12.443 | similar to UniRef100_A9SNZ3 Cluster: Predicted protein; n=1; Physcomitrella patens subsp. patens\|Rep: Predicted protein - Physcomitrella patens subsp. patens, partial (3%) |
|  | CTTTCCCAATCCACCCATGC |  |  |  |  |
|  | CTTTCCCAATCCACCCATGCC |  |  |  |  |
|  | TCCCAATCCACCCATGCC |  |  |  |  |
|  | TCTTTCCCAATCCACCCA |  |  |  |  |
|  | TCTTTCCCAATCCACCCAT |  |  |  |  |
|  | TCTTTCCCAATCCACCCATG | TC17908 | 2.5 | 17.071 | homologue to UniRef100_A7PCU9 Cluster: Chromosome chr17 scaffold_12, whole genome shotgun sequence; n=1; Vitis vinifera\|Rep: Chromosome chr17 scaffold_12, whole genome shotgun sequence - Vitis vinifera (Grape), partial (21%) |
|  | TCTTTCCCAATCCACCCATGC | TC17908 | 2.5 | 17.071 | homologue to UniRef100_A7PCU9 Cluster: Chromosome chr17 scaffold_12, whole genome shotgun sequence; n=1; Vitis vinifera\|Rep: Chromosome chr17 scaffold_12, whole genome shotgun sequence - Vitis vinifera (Grape), partial (21%) |
|  | TCTTTCCCAATCCACCCATGCC | TC17908 | 2.5 | 17.071 | homologue to UniRef100_A7PCU9 Cluster: Chromosome chr17 scaffold_12, whole genome shotgun sequence; n=1; Vitis vinifera\|Rep: Chromosome chr17 scaffold_12, whole genome shotgun sequence - Vitis vinifera (Grape), partial (21%) |
|  | TCTTTCCCAATCCACCCATGCCT | TC17908 | 2.5 | 17.071 | homologue to UniRef100_A7PCU9 Cluster: Chromosome chr17 scaffold_12, whole genome shotgun sequence; n=1; Vitis vinifera\|Rep: Chromosome chr17 scaffold_12, whole genome shotgun sequence - Vitis vinifera (Grape), partial (21%) |
|  | TGGCATGGGTAAGTGGGGAAGA | AM289150 | 2 | 24.492 | similar to UniRef100_A7Q7U0 Cluster: Chromosome chr18 scaffold_61, whole genome shotgun sequence; n=1; Vitis vinifera\|Rep: Chromosome chr18 scaffold_61, whole genome shotgun sequence - Vitis vinifera (Grape), partial (33%) |
|  | TTAGGTTTCCTCTTATTCATCC | DW345294 | 2.5 | 11.235 | similar to UniRef100_Q8LK65 Cluster: DNA-binding protein phosphatase 2C; n=1; Nicotiana tabacum\|Rep: DNA-binding protein phosphatase 2C - Nicotiana tabacum (Common tobacco), partial (38%) |
|  | TTCCCAATCCACCCATGCCT |  |  |  |  |
|  | TTCCCAATCCACCCATGCCTT |  |  |  |  |
|  | TTTCCCAATCCACCCATGCCT |  |  |  |  |
|  | TTTCCCAATCCACCCATGCCTT |  |  |  |  |
|  | TTTCCCAATCCACCCATGCCTTA |  |  |  |  |
|  | TTTCCTCTTATTCATCCCTCT | TC12029 | 2.5 | 14.849 | similar to UniRef100_Q852S9 Cluster: S-adenosylmethionine decarboxylase; n=1; Malus x domestica\|Rep: S-adenosylmethionine decarboxylase - Malus domestica (Apple) (Malus sylvestris), complete |
|  |  |  |  |  |  |
| **7_23** | AAGAAAGCTGTGGGAGAACAT | TC14752 | 1.5 | 10.169 | homologue to UniRef100_A7USG3 Cluster: AGAP000586-PA; n=1; Anopheles gambiae str. PEST\|Rep: AGAP000586-PA - Anopheles gambiae str. PEST, partial (16%) |
|  | AAGAAAGCTGTGGGAGAACATGGC | TC14752 | 1.5 | 10.169 | homologue to UniRef100_A7USG3 Cluster: AGAP000586-PA; n=1; Anopheles gambiae str. PEST\|Rep: AGAP000586-PA - Anopheles gambiae str. PEST, partial (16%) |
|  | CACAGCTTTCTTGAACTT |  |  |  |  |
|  | CCACAGCTTTCTTGAACT |  |  |  |  |
|  | CTCAAGAAAGCTGTGGGAGA | TC14752 | 1.5 | 15.759 | homologue to UniRef100_A7USG3 Cluster: AGAP000586-PA; n=1; Anopheles gambiae str. PEST\|Rep: AGAP000586-PA - Anopheles gambiae str. PEST, partial (16%) |
|  | GCTCAAGAAAGCTGTGGGAGA | TC14752 | 2 | 15.806 | homologue to UniRef100_A7USG3 Cluster: AGAP000586-PA; n=1; Anopheles gambiae str. PEST\|Rep: AGAP000586-PA - Anopheles gambiae str. PEST, partial (16%) |
|  | TATAAACAAGTCCTGGTCATGCTT | DW342520 | 3 | 21.073 |  |
|  | TCCACAGCTTTCTTGAACT |  |  |  |  |
|  | TCCACAGCTTTCTTGAACTT | TC12841 | 3 | 14.24 | similar to UniRef100_Q9LQU1 Cluster: F10B6.30; n=1; Arabidopsis thaliana\|Rep: F10B6.30 - Arabidopsis thaliana (Mouse-ear cress), partial (43%) |
|  | TTCCACAGCTTTCTTGAA |  |  |  |  |
|  | TTCCACAGCTTTCTTGAAC |  |  |  |  |
|  | TTCCACAGCTTTCTTGAACT | TC16190 | 2.5 | 17.373 | similar to UniRef100_A7QGZ2 Cluster: Chromosome chr3 scaffold_95, whole genome shotgun sequence; n=1; Vitis vinifera\|Rep: Chromosome chr3 scaffold_95, whole genome shotgun sequence - Vitis vinifera (Grape), partial (47%) |
|  | TTCCACAGCTTTCTTGAACTT | TC16190 | 2.5 | 17.373 | similar to UniRef100_A7QGZ2 Cluster: Chromosome chr3 scaffold_95, whole genome shotgun sequence; n=1; Vitis vinifera\|Rep: Chromosome chr3 scaffold_95, whole genome shotgun sequence - Vitis vinifera (Grape), partial (47%) |
|  | TTCCACAGCTTTCTTGAACTTC | TC16190 | 2.5 | 17.373 | similar to UniRef100_A7QGZ2 Cluster: Chromosome chr3 scaffold_95, whole genome shotgun sequence; n=1; Vitis vinifera\|Rep: Chromosome chr3 scaffold_95, whole genome shotgun sequence - Vitis vinifera (Grape), partial (47%) |
|  |  |  |  |  |  |
| **7_24** | ACACTGTGGCTCGTTGTGTTGTCA | FC863901 | 3 | 15.259 | homologue to UniRef100_A1WDA9 Cluster: Potassium efflux system protein precursor; n=1; Acidovorax sp. JS42\|Rep: Potassium efflux, partial (2%) |
|  | ACGTTATGTTGTCAAATTGTC | DW344564 | 2.5 | 4.64 |  |
|  | ATGTTGTCAAATTGTCAATC | DW341744 | 1 | 14.307 | UniRef100_Q76IJ7 Cluster: High-affinity nitrate transporter; n=1; Prunus persica\|Rep: High-affinity nitrate transporter - Prunus persica (Peach), partial (22%) |
|  | CAACGTGACAACACAACGAGC |  |  |  |  |
|  | CAACGTGACAACACAACGAGCC |  |  |  |  |
|  | CACGTTATGTTGTCAAATTGTC | DW341744 | 2.5 | 15.544 | UniRef100_Q76IJ7 Cluster: High-affinity nitrate transporter; n=1; Prunus persica\|Rep: High-affinity nitrate transporter - Prunus persica (Peach), partial (22%) |
|  | TATGTTGTCAAATTGTCAAT | DW341744 | 1 | 14.239 | UniRef100_Q76IJ7 Cluster: High-affinity nitrate transporter; n=1; Prunus persica\|Rep: High-affinity nitrate transporter - Prunus persica (Peach), partial (22%) |
|  | TATGTTGTCAAATTGTCAATC | DW341744 | 1 | 14.239 | UniRef100_Q76IJ7 Cluster: High-affinity nitrate transporter; n=1; Prunus persica\|Rep: High-affinity nitrate transporter - Prunus persica (Peach), partial (22%) |
|  | TGAACACAAAGATACATGCCCG |  |  |  |  |
|  | TTGACAACGTGACAACACAAC |  |  |  |  |
|  |  |  |  |  |  |
| **7_25** | GACAGAAGAGAGTGAGCAC |  |  |  |  |
|  | GCTCACTTCTCTCTCTGTCAGC | AJ872631 | 3 | 4.928 | similar to UniRef100_A9V087 Cluster: Predicted protein; n=1; Monosiga brevicollis MX1\|Rep: Predicted protein - Monosiga brevicollis MX1, partial (3%) |
|  | TGACAGAAGAGAGTGAGCA |  |  |  |  |
|  | TGACAGAAGAGAGTGAGCAC | DY638783 | 3 | 18.898 | similar to UniRef100_A7NXB7 Cluster: Chromosome chr5 scaffold_2, whole genome shotgun sequence; n=1; Vitis vinifera\|Rep: Chromosome chr5 scaffold_2, whole genome shotgun sequence - Vitis vinifera (Grape), partial (79%) |
|  | TGACAGAAGAGAGTGAGCACA | DY638783 | 3 | 18.898 | similar to UniRef100_A7NXB7 Cluster: Chromosome chr5 scaffold_2, whole genome shotgun sequence; n=1; Vitis vinifera\|Rep: Chromosome chr5 scaffold_2, whole genome shotgun sequence - Vitis vinifera (Grape), partial (79%) |
|  |  |  |  |  |  |
| **8_16** | CCGACAAGCGTGCTCTCTCTCGTT | TC16877 | 3 | 20.195 | homologue to gb\|AF036494.1\|AF036494 Eucryphia lucida large subunit 26S ribosomal RNA gene, partial sequence, partial (20%) |
|  | GTGCTCTCTCTTGTTGTCATG | TC11306 | 2.5 | 18.627 | similar to UniRef100_A7QIY1 Cluster: Chromosome chr2 scaffold_105, whole genome shotgun sequence; n=1; Vitis vinifera\|Rep: Chromosome chr2 scaffold_105, whole genome shotgun sequence - Vitis vinifera (Grape), partial (77%) |
|  | TGACAACGAGAGAGAGCAC |  |  |  |  |
|  | TGACAACGAGAGAGAGCACG | AJ827655 | 3 | 8.944 | similar to UniRef100_Q9LND1 Cluster: Ethylene-responsive transcription factor ERF094; n=1; Arabidopsis thaliana\|Rep: Ethylene-responsive transcription factor ERF094 - Arabidopsis thaliana (Mouse-ear cress), partial (11%) |
|  | TGACAACGAGAGAGAGCACGC | AJ827655 | 3 | 8.944 | similar to UniRef100_Q9LND1 Cluster: Ethylene-responsive transcription factor ERF094; n=1; Arabidopsis thaliana\|Rep: Ethylene-responsive transcription factor ERF094 - Arabidopsis thaliana (Mouse-ear cress), partial (11%) |
|  | TTGACAACGAGAGAGAGCAC | AJ827655 | 2.5 | 8.951 | similar to UniRef100_Q9LND1 Cluster: Ethylene-responsive transcription factor ERF094; n=1; Arabidopsis thaliana\|Rep: Ethylene-responsive transcription factor ERF094 - Arabidopsis thaliana (Mouse-ear cress), partial (11%) |
|  | TTGACAACGAGAGAGAGCACG | AJ827655 | 2.5 | 8.951 | similar to UniRef100_Q9LND1 Cluster: Ethylene-responsive transcription factor ERF094; n=1; Arabidopsis thaliana\|Rep: Ethylene-responsive transcription factor ERF094 - Arabidopsis thaliana (Mouse-ear cress), partial (11%) |
|  | TTGACAACGAGAGAGAGCACGC | AJ827655 | 2.5 | 8.951 | similar to UniRef100_Q9LND1 Cluster: Ethylene-responsive transcription factor ERF094; n=1; Arabidopsis thaliana\|Rep: Ethylene-responsive transcription factor ERF094 - Arabidopsis thaliana (Mouse-ear cress), partial (11%) |
|  | TTGTCGGCACCCATGAAAGGGCCA |  |  |  |  |
|  | TTTGACAACGAGAGAGAGCAC | AJ827655 | 2.5 | 8.934 | similar to UniRef100_Q9LND1 Cluster: Ethylene-responsive transcription factor ERF094; n=1; Arabidopsis thaliana\|Rep: Ethylene-responsive transcription factor ERF094 - Arabidopsis thaliana (Mouse-ear cress), partial (11%) |
|  |  |  |  |  |  |
| **8_19** | AATGTCGTCTGGTTCGAGA |  |  |  |  |
|  | AATGTCGTCTGGTTCGAGATC | DW341237 | 3 | 13.673 | similar to UniRef100_A7QN82 Cluster: Chromosome undetermined scaffold_131, whole genome shotgun sequence; n=1; Vitis vinifera\|Rep: Chromosome undetermined scaffold_131, whole genome shotgun sequence - Vitis vinifera (Grape), partial (58%) |
|  | ATTTCGGACCAGGCTTCATTC |  |  |  |  |
|  | ATTTCGGACCAGGCTTCATTCCCC |  |  |  |  |
|  | CGGACCAGGCTTCATTCC |  |  |  |  |
|  | CGGACCAGGCTTCATTCCC |  |  |  |  |
|  | CGGACCAGGCTTCATTCCCC | FC862354 | 3 | 17.607 | weakly similar to UniRef100_Q93ZH4 Cluster: AT5g66850/MUD21_11; n=1; Arabidopsis thaliana\|Rep: AT5g66850/MUD21_11 - Arabidopsis thaliana (Mouse-ear cress), partial (9%) |
|  | CGGACCAGGCTTCATTCCCCT | FC862354 | 3 | 17.607 | weakly similar to UniRef100_Q93ZH4 Cluster: AT5g66850/MUD21_11; n=1; Arabidopsis thaliana\|Rep: AT5g66850/MUD21_11 - Arabidopsis thaliana (Mouse-ear cress), partial (9%) |
|  | GAATGTCGTCTGGTTCGAGA | TC10918 | 3 | 14.697 | similar to UniRef100_Q9M0H8 Cluster: Predicted proline-rich protein; n=1; Arabidopsis thaliana\|Rep: Predicted proline-rich protein - Arabidopsis thaliana (Mouse-ear cress), partial (31%) |
|  | GACCAGGCTTCATTCCCC |  |  |  |  |
|  | GACCAGGCTTCATTCCCCTCA | DW345813 | 2 | 23.095 | similar to UniRef100_A7Q8Q2 Cluster: Chromosome chr5 scaffold_64, whole genome shotgun sequence; n=1; Vitis vinifera\|Rep: Chromosome chr5 scaffold_64, whole genome shotgun sequence - Vitis vinifera (Grape), partial (9%) |
|  | GATTTCGGACCAGGCTTCATTCCC |  |  |  |  |
|  | GGAATGTCGTCTGGTTCGA |  |  |  |  |
|  | GGAATGTCGTCTGGTTCGAGA |  |  |  |  |
|  | GGAATGTCGTCTGGTTCGAGAT |  |  |  |  |
|  | GGACCAGGCTTCATTCCC |  |  |  |  |
|  | GGACCAGGCTTCATTCCCC |  |  |  |  |
|  | GGGAATGTCGTCTGGTTCGAG | TC10918 | 3 | 14.859 | similar to UniRef100_Q9M0H8 Cluster: Predicted proline-rich protein; n=1; Arabidopsis thaliana\|Rep: Predicted proline-rich protein - Arabidopsis thaliana (Mouse-ear cress), partial (31%) |
|  | TCGGACCAGGCTTCATTC |  |  |  |  |
|  | TCGGACCAGGCTTCATTCC |  |  |  |  |
|  | TCGGACCAGGCTTCATTCCC |  |  |  |  |
|  | TCGGACCAGGCTTCATTCCCC |  |  |  |  |
|  | TCGGACCAGGCTTCATTCCCCT |  |  |  |  |
|  | TCGGACCAGGCTTCATTCCCCTC |  |  |  |  |
|  | TTCGGACCAGGCTTCATTCC |  |  |  |  |
|  | TTCGGACCAGGCTTCATTCCC |  |  |  |  |
|  | TTCGGACCAGGCTTCATTCCCC |  |  |  |  |
|  | TTCGGACCAGGCTTCATTCCCCT |  |  |  |  |
|  | TTGAGGGGAATGTCGTCTGG |  |  |  |  |
|  | TTTCGGACCAGGCTTCATTCC |  |  |  |  |
|  |  |  |  |  |  |
| **8_21** | CACGTGCTCCCCTTCTCC |  |  |  |  |
|  | CACGTGCTCCCCTTCTCCAAC |  |  |  |  |
|  | TGGAGAAGCAGGGCACGTGCA | TC12261 | 2.5 | 11.498 | similar to UniRef100_A7PIJ8 Cluster: Chromosome chr13 scaffold_17, whole genome shotgun sequence; n=1; Vitis vinifera\|Rep: Chromosome chr13 scaffold_17, whole genome shotgun sequence - Vitis vinifera (Grape), partial (33%) |
